# iVikodak—A Platform and Standard Workflow for Inferring, Analyzing, Comparing, and Visualizing the Functional Potential of Microbial Communities

**DOI:** 10.3389/fmicb.2018.03336

**Published:** 2019-01-14

**Authors:** Sunil Nagpal, Mohammed Monzoorul Haque, Rashmi Singh, Sharmila S. Mande

**Affiliations:** Bio-Sciences R&D Division, TCS Research, Tata Consultancy Services, Pune, India

**Keywords:** inferred functions, 16S metagenome, functional metagenomics, functions of microbial communities, microbiome analysis, visualization, data analyses

## Abstract

**Background:** The objectives of any metagenomic study typically include identification of resident microbes and their relative proportions (taxonomic analysis), profiling functional diversity (functional analysis), and comparing the identified microbes and functions with available metadata (comparative metagenomics). Given the advantage of cost-effectiveness and convenient data-size, amplicon-based sequencing has remained the technology of choice for exploring phylogenetic diversity of an environment. A recent school of thought, employing the existing genome annotation information for inferring functional capacity of an identified microbiome community, has given a promising alternative to Whole Genome Shotgun sequencing for functional analysis. Although a handful of tools are currently available for function inference, their scope, functionality and utility has essentially remained limited. Need for a comprehensive framework that expands upon the existing scope and enables a standardized workflow for function inference, analysis, and visualization, is therefore felt.

**Methods:** We present iVikodak, a multi-modular web-platform that hosts a logically inter-connected repertoire of functional inference and analysis tools, coupled with a comprehensive visualization interface. iVikodak is equipped with microbial co-inhabitance pattern driven published algorithms along with multiple updated databases of various curated microbe-function maps. It also features an advanced task management and result sharing system through introduction of personalized and portable dashboards.

**Results:** In addition to inferring functions from 16S rRNA gene data, iVikodak enables (a) an in-depth analysis of specific functions of interest (b) identification of microbes contributing to various functions (c) microbial interaction patterns through function-driven correlation networks, and (d) simultaneous functional comparison between multiple microbial communities. We have bench-marked iVikodak through multiple case studies and comparisons with existing state of art. We also introduce the concept of a public repository which provides a first of its kind community-driven framework for scientific data analytics, collaboration and sharing in this area of microbiome research.

**Conclusion:** Developed using modern design and task management practices, iVikodak provides a multi-modular, yet inter-operable, one-stop framework, that intends to simplify the entire approach toward inferred function analysis. It is anticipated to serve as a significant value addition to the existing space of functional metagenomics.

iVikodak web-server may be freely accessed at https://web.rniapps.net/iVikodak/.

## Introduction

Whole Genome Shotgun (WGS) metagenomic DNA sequencing (and subsequent computational analysis of resultant sequence data) helps in not only profiling or cataloging the (microbial) biodiversity characterizing a given habitat, but also enables estimation (of the types and proportions) of various biological functions encoded within the genetic material of microbes resident in that ecological niche (Quince et al., 2017). Multitude of tools and analytical workflows currently exist for WGS driven integrated metagenomics (Keegan et al., 2016; Narayanasamy et al., 2016; White et al., 2017). However, due to high sequencing (and significant downstream computational) costs associated with WGS approach (Bose et al., 2015; Quince et al., 2017; Rossen et al., 2018), initial exploration and estimation of microbial biodiversity (of an environment of interest) is done, in most cases, using amplicon sequencing (Petrosino et al., 2009; Ganju et al., 2016). The latter approach involves PCR amplification and sequencing of a taxonomically informative target genomic marker (e.g., 16S rRNA gene) from the DNA extracted from all microbes present in a given environmental sample. The primary objective of the aforesaid (16S rRNA gene) amplicon-based sequencing has therefore been limited to obtaining quick snap-shots of microbial taxonomic diversity in a cost-effective manner. A plethora of bioinformatics tools and standard workflows/ pipelines are currently available for pre-processing and analysing such amplicon sequencing (16S rRNA gene) datasets to meet the said objectives of taxonomic profiling and analyses (Kuczynski et al., 2011; Arndt et al., 2012; McMurdie and Holmes, 2013; Zakrzewski et al., 2017).

A recent school of thought however adds a new dimension to the utility of amplicon sequencing, i.e., “inferring” functional potential of microbial communities “from taxonomic abundance profiles” (Langille et al., 2013). Such inferences are based on the assumption that the pool of genes (and associated functions) in a given microbial community is ultimately a function of the “types and relative abundances” of various microbes (constituting that community). Consequently, given a quantified taxonomic profile corresponding to a given microbial community (residing in a particular environmental niche), it is possible to estimate the functional potential encoded by various microbes constituting the said environment.

A handful of recent methods like PICRUSt (Langille et al., 2013), Tax4Fun (Aßhauer et al., 2015), and Vikodak (Nagpal et al., 2016), have successfully exploited the above mentioned taxa-function inter-relationship for inferring (*in silico*) the functional capacity of microbial communities from their taxonomic profiles (generated through amplicon sequencing). The mentioned methods use distinct algorithms to infer or predict abundances of various functions for a given metagenomic environment in the form of “function abundance matrices.” Given that this school of thought is a recent development, the avenue of probing the functional capacity of a metagenomic environment using (16S rRNA gene) amplicon sequencing, has still remained limited to generation of mere “textual matrices” representing the functional abundance for each sample of an environment. A lot of “potential scope” remains unexplored and a standard workflow in the domain of amplicon sequencing driven functional metagenomic analysis is currently lacking. For example, given the availability of various information rich databases like IMG (Markowitz et al., 2012), PATRIC (Wattam et al., 2014), KEGG (Kanehisa and Goto, 2000), that hold prior-collated information about genome-specific functional potentials, it is possible to infer functional correlation based microbe-microbe interaction patterns for a given environment. In addition, functional analysis can also be explored at a granular level to deliver “single pathway or module specific” insights to the researchers. Furthermore, given the availability of established statistical tools and visualization technologies, coupled with multivariate nature of inferred function data, it is possible to define a logical workflow that can not only infer functions, but also perform meta-analyses, statistical comparisons, deep probing and generate meaningful and insightful visualizations.

In the above context, it may be noted that performing such meaningful analysis necessitates end users to garner working knowledge of not only available state-of-art “function prediction” tools (and the input/ output formats, run-time parameters they support), but also an array of statistical and visualization tools, which are additionally required to be implemented for efficient downstream analysis, and hence realize the said workflow for this domain. Needless to say, a platform that enables and (more importantly) automates all of the above mentioned steps is expected to greatly ease the burden on end users. Access to such a framework would enable researchers to efficiently focus on deriving and analysing “functional” insights and subsequently scrutinize the observed trends with respect to related “metadata” and observed taxonomic variation. Such an automated framework would effectively relieve researchers of the mundane nitty-gritty's of (input/ output) data parsing, processing, and visualization support required at almost every stage of analysis, in addition to providing more scope for functional exploration.

Although, the idea of integrating a compendium of tools and utilities to develop such an automated one-stop “infer-compare-visualize” frame-work typically appears more of an e-enablement exercise (driven by IT expertise), it is important to note that the real value add of any such “16S rRNA gene sequencing based” functional annotation framework lies not only in terms of the variety of domain information and functionalities it provides, but also the types of “biologically-relevant” insights it enables for obtaining possible and meaningful answers. The said information, functionalities, and insights may be in terms of (a) *Back-end database*: with respect to the variety, accuracy, and comprehensiveness of its back-end functional-unit (cross)-mappings (b) *Algorithms*: in terms of the types of algorithms and the functional assumptions these algorithms enable for eliciting biologically relevant functional inference(s) (c) *Taxa-function inter-relationships*: in terms of flexibility to back-trace (and visualize in context) the taxonomic sources of the inferred functions and/or deeply probing a pathway or function of interest (d) *Function contribution-based taxonomic relationships/network identification*: derived through co-relation analysis of (inferred) functional capabilities of contributing microbes. Such analysis are expected to enable endusers to additionally narrow down upon, at a microbial (sub) community level, the specific taxonomic drivers behind observed functional patterns or shifts.

**Figure 1:**
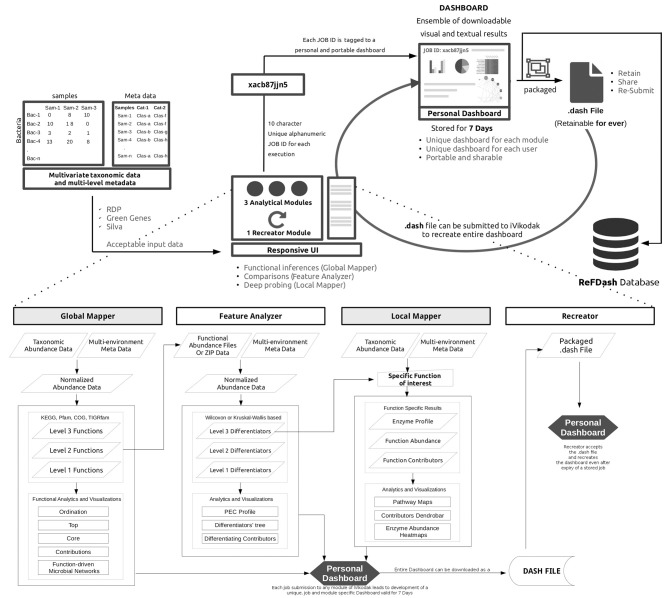
Overview of iVikodak web platform and its structured workflow. An overview of iVikodak's task management and well-structured personalized approach toward 16s rRNA gene sequencing based functional inference, analyses and visualization. Three inter-connected modules of iVikodak ensure comprehensive and meaningful analyses. Each submission to (any module of) iVikodak is tagged to a unique JOB ID, which provides access to a personal and portable dashboard. A dashboard represents an ensemble of “analysable, analyzed, and visualized” results, specific to the chosen module and the type of data uploaded by the end user. The generated dashboards (and associated taxonomic/functional data) can be deposited to the ReFDash repository. This is expected to pave the way for building/populating a community-driven readily accessible database of amplicon sequencing-based functional metagenomics projects and associated data.

Considering the existing state of art in the amplicon sequencing driven functional metagenomic space, we present “iVikodak”—a multi-modular, yet, inter-operable web application framework that provides end users algorithmic options (coupled with updated back-end domain information) for comprehensively inferring the functional potential of microbial communities. At the outset it may be noted that iVikodak represents a significantly upgraded version of Vikodak (Nagpal et al., 2016). iVikodak vastly advances upon the scope and variety of functionalities provided not only in Vikodak, but also other available tools (Langille et al., 2013; Aßhauer et al., 2015; McNally et al., 2018) in the said space. Various functionalities in iVikodak have been intuitively designed and have been e-enabled in formats that simplify for end users the entire approach toward inferred function analysis. Advancements in iVikodak are not limited to the variety of available options for statistical and visual analyses, but also from a user interface (UI) and user-experience (UX) perspective, through development of a well-structured task and data management system. The concept of a public repository (named “ReFDash”) which, in addition to hosting pre-generated functional profiles of various environments, provides the research community a frame-work for scientific data collaboration/sharing is also introduced. Figure 1 provides an overview of iVikodak's functionalities and workflow. Subsequent sections of this paper provide a detailed description of various features of the iVikodak platform. Case-studies highlighting the comparisons with other tools, and utility of specific technical advancements and visualizations incorporated in iVikodak are also provided.

## Results and Discussion

### A Comparison of iVikodak With Available Tools and Platforms

As a one-stop “infer-compare-visualize” automated frame-work, iVikodak represents a significant advancement over the textual function abundance matrices generated by the first generation of function inference/prediction tools viz. PICRUSt (Langille et al., 2013), Tax4Fun (Aßhauer et al., 2015), and Vikodak (Nagpal et al., 2016). The prime role of the latter tools is to infer, from end user provided taxonomic profiles, the principal building blocks i.e., information about the types and abundances of various functional units. In an ideal scenario, end users would prefer access to such an inferred or predicted information in terms of varied types of possible functional units (as many as possible). iVikodak, with its updated back-end database (with comprehensive functional unit cross-mapping information) provides, by default, functional annotations in terms of, enzyme copy numbers (EC) (McDonald et al., 2015), TIGRfam (Haft et al., 2003), Pfam (Finn et al., 2014), and COG (Tatusov et al., 2000) categories. Although PICRUSt provides a similar repertoire of information (albeit as textual outputs), iVikodak scores over PICRUSt (and the other two tools) by enabling end users to intuitively compare, probe, and visualize this wealth of information. For instance, the simple automated utility wherein end users are provided queryable tables that not only indicate the subset of bacteria contributing to a chosen function of interest, but also an intuitive (and interactive) visual interface that helps in simultaneously examining the taxonomic and functional context of the same.

In this specific context of comparing iVikodak with other tools of the same genre, it may be noted that a recently published web-utility “Burrito” (McNally et al., 2018) also provides an analogous visualization layout for analysing taxa–function relationships. Using Burrito, users can browse, interactively explore and/or visualize the proportions of individual functions across various samples, along-side the taxa contributing to the said functions. Although Burrito, as a tool, does provide for an automated functional inference cum visualization frame-work besides hosting a decent set of backend parsers and related frontend utilities, it falls short in terms of the following important aspects. Primarily, Burrito's interface is limited to displaying 3 distinct types of visualizations. Prominently, “bar-plot” representations of predicted functions are provided along-side expandable/collapsible “cladograms” that represent corresponding taxonomic hierarchies. These layouts merely enable end users to interactively visualize the types and abundances of various functions (inferred using PICRUSt) in the context of their source taxonomy. In contrast, iVikodak provides end users a comprehensive and inter-operable framework comprised of three logically connected modules, each one of them in turn, having their own repertoire of utilities and multiple types of visualizations. To highlight the array of differences between iVikodak and Burrito, we have used the same dataset (Theriot et al., 2014) that was previously utilized in Burrito for showcasing/ exemplifying its functionalities. This comparison (case study 1) throws light not only on the functionalities available in iVikodak, but also attempts to put forward the vast range of functionalities that were hitherto unavailable (or are not comprehensive enough) in other existing analogous tools including Burrito. Table 1 provides a comparison of features available in iVikodak, Burrito, Vikodak, PICRUSt and Tax4Fun.

**Table 1:** Comparison of iVikodak with other function inference tools.

**Features**	**iVikodak**	**Burrito**	**Vikodak**	**PICRUSt**	**Tax4Fun**
Multivariate inputs	✓	✓	✓	✓	✓
Default RDP compatibility	✓	**×**	✓	**×**	✓
Default Greengenes compatibility	✓	✓	**×**	✓	**×**
Default SILVA compatibility	✓	**×**	✓	**×**	✓
KEGG, Pfam, COG, TIGRfam inference	✓	✓	**×**	✓	**×**
In-depth analysis of pathway of interest	✓	**×**	✓	**×**	**×**
Co-inhabitance based algorithms	✓	**×**	✓	**×**	**×**
Gene Quorum Assumption	✓	**×**	✓	**×**	**×**
Metadata acceptance	✓	✓	✓	**×**	**×**
Multiple Categories of Metadata acceptance	✓	**×**	✓	**×**	**×**
Tools for Statistical Comparisons	✓	✓	**×**	**×**	**×**
Graphical Visualizations	✓	✓	**×**	**×**	**×**
- Ordination (PCoA)	✓	**×**	**×**	**×**	**×**
- Core Functions (Heatmaps)	✓	**×**	**×**	**×**	**×**
- Top Functions (Grouped Box plots)	✓	**×**	**×**	**×**	**×**
- Top Functions (Grouped Bar plots)	**×**	✓	**×**	**×**	**×**
- Differentiating functions (Heatmaps)	✓	**×**	**×**	**×**	**×**
- Differentiating functions (Cladograms)	✓	**×**	**×**	**×**	**×**
- Taxa – Function Contribution tree	✓	✓	**×**	**×**	**×**
- Function-driven Networks	✓	**×**	**×**	**×**	**×**
- Enzyme Abundance Profiles (Heatmaps)	✓	**×**	**×**	**×**	**×**
3D and Colored KEGG Pathway Maps	✓	**×**	**×**	**×**	**×**
Task Management (multi-jobs, JOB IDs etc.)	✓	**×**	**×**	**×**	**×**
Personalized and Portable Dashboards	✓	**×**	**×**	**×**	**×**

### Functionalities Enabling Generation and Visualization of Biologically Meaningful Insights

The visual options provided in iVikodak are not to be construed as a mere e-enablement exercise. For instance, the “*functional networks*” generated (from each of the taxonomic profiles corresponding to one or multiple environments) by the *Global Mapper module* are based on the assumption that microbes co-contributing to specific functions (in a correlated manner) are likely to be interacting (details of various modules provided in section Methods, Modules, and Functionalities). Enabling (automated) generation and (interactive) visualization of such networks (and their properties) can potentially help end users in identifying microbial sub-communities that are likely drivers of functional shifts observed between two or more environments. Furthermore, the interactive PCoA ordination plots (in 2D and 3D formats) and the accompanying bar-charts (which visually indicate the proportion of samples in each of the clusters generated during ordination) represent another such functionality (and automated utility) of iVikodak (which tools like Burrito do not provide). It may be noted that during ordination (in iVikodak), samples are clustered based on their (inferred) functional potential, and not as per taxonomic proportions mentioned in the uploaded input profiles.

From an e-enablement perspective, the *range of visualizations* in iVikodak's Global Mapper Module is worth mentioning. “Box-plots” representing abundance of “*top*” (i.e., most abundant) functions, “heat-maps” of functions identified as “*core*” to given environments, 2D/3D ordination plots, function-contribution-based networks are some of the utilities that iVikodak provides. The highlight is that end users can overlay as many types of (available) metadata features over the entire repertoire of visualizations to analyse and download relevant (publication-friendly) images for scientific sharing. Unlike existing tools, right from the step of uploading input data, iVikodak tends to reduce the pre-processing efforts that are typically required to be done by end users. The acceptance of taxonomic profile data generated using any of the three popular taxonomic classification frame-works is one such example.

Apart from identifying and visualizing at various *p*-value thresholds, the set of pathways (or pathway-classes) whose abundances are found to have a statistically significant difference (between two or more environments), the *PEC profile chart* generated by the *inter sample feature analyser (ISFA) module* represents another important functionality that adds value from a biological viewpoint (details in section Methods, Modules, and Functionalities). During the backend process of functional inference, besides providing unfiltered results, iVikodak additionally reports a pathway to be “present” (in a given environment) only when the proportion or the number of its inferred constituent enzymes exceeds at least a minimum quorum of 50%. This threshold is referred to as the “Pathway Exclusion Cut-off” (PEC) threshold (Nagpal et al., 2016). Given that a different functional context may necessitate end users to employ threshold(s) higher than the 50% minimum, iVikodak performs these computations at 5 progressively higher PEC levels, viz. 50, 60, 70, 80, and 90%, and provides a consensus PEC profile chart (in form of a heat-map). This enables users to visually take an informed decision regarding the set of “differentiating” pathways to finally consider (or purge) from their final analysis.

The *local mapper* is a unique module that sets apart iVikodak in comparison to its peers. By enabling end users intending to drill their analysis to the level of individual functional units that constitute a specific pathway of interest, this module serves as a logical extension to the other two modules of iVikodak (details of the module are provided in section Methods, Modules, and Functionalities). The module provides a contextual platform that facilitates visual analysis of the presence and abundance of various enzymes constituting a given pathway of interest. Typically, end users can employ this module to probe (at a high level of granularity) one or more pathways identified by ISFA module to have a significantly different abundance pattern between the compared environments (e.g., healthy vs. diseased, time-series data, etc.). The facility to generate 3D formatted and colored pathway map(s) is expected to vastly improve the visual (analysis) experience for end users.

### Case Study 1: Temporal Observation of Functional Perturbations in Gut Microbiota of Antibiotic Treated Mice

This case-study pertains to available gut microbiome data (from case and time-matched controls) obtained in a longitudinal fashion from mice administered with antibiotics (Theriot et al., 2014). The datasets corresponded to samples obtained at 2 days and at 6 weeks post-treatment with antibiotics (indicated as Abx_Day2, Abx_Day42 for treated samples and Control_Day2, Control_Day42 for control samples).

**Figure 2:**
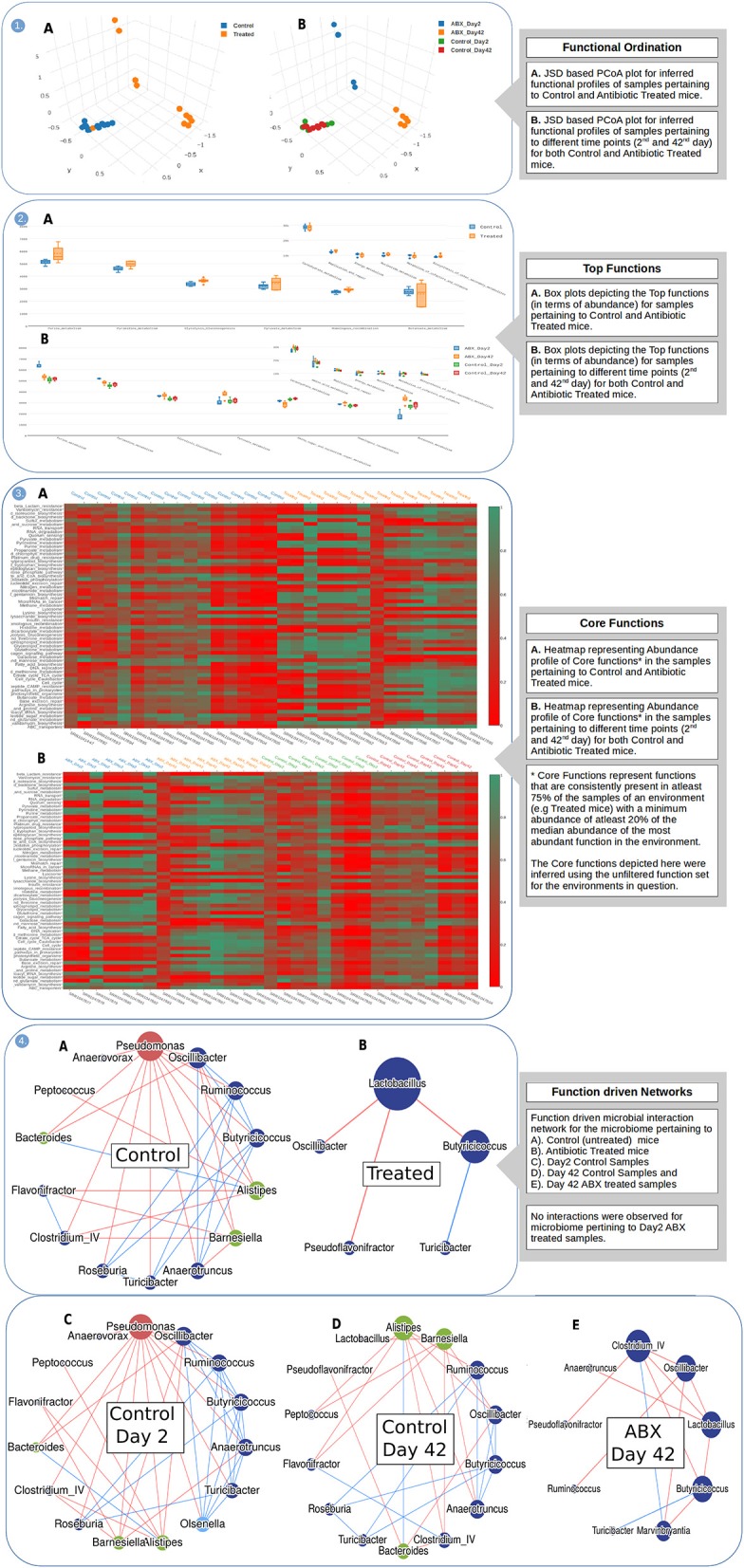
Results of iVikodak's Global Mapper Module for datasets corresponding to case study 1. Plots represent (1) Ordination (2) Top Functions (3) Core Functions (4) Function driven networks aimed at temporal observation of inferred “functional perturbations” in gut microbiota of antibiotic treated mice.

**Figure 3:**
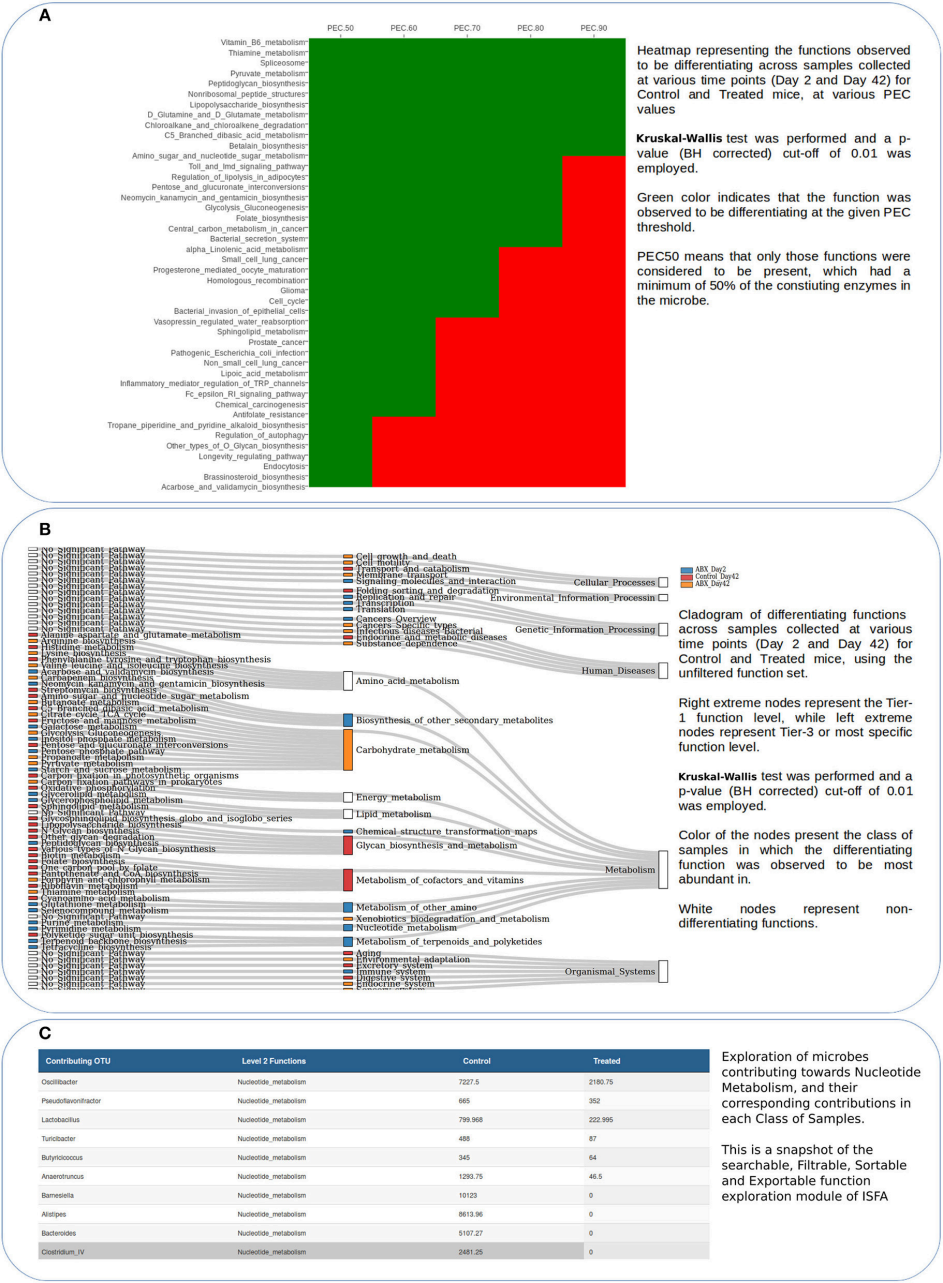
Results of iVikodak's ISFA Module for datasets corresponding to case study 1. Plots represent **(A)** PEC Profile of differentiating functions **(B)** Sankey based cladogram of differentiating functions **(C)** Contributors' profile for differenatiating functions, aimed at temporal observation of inferred “functional perturbations” in gut microbiota of antibiotic treated mice.

**Figure 4:**
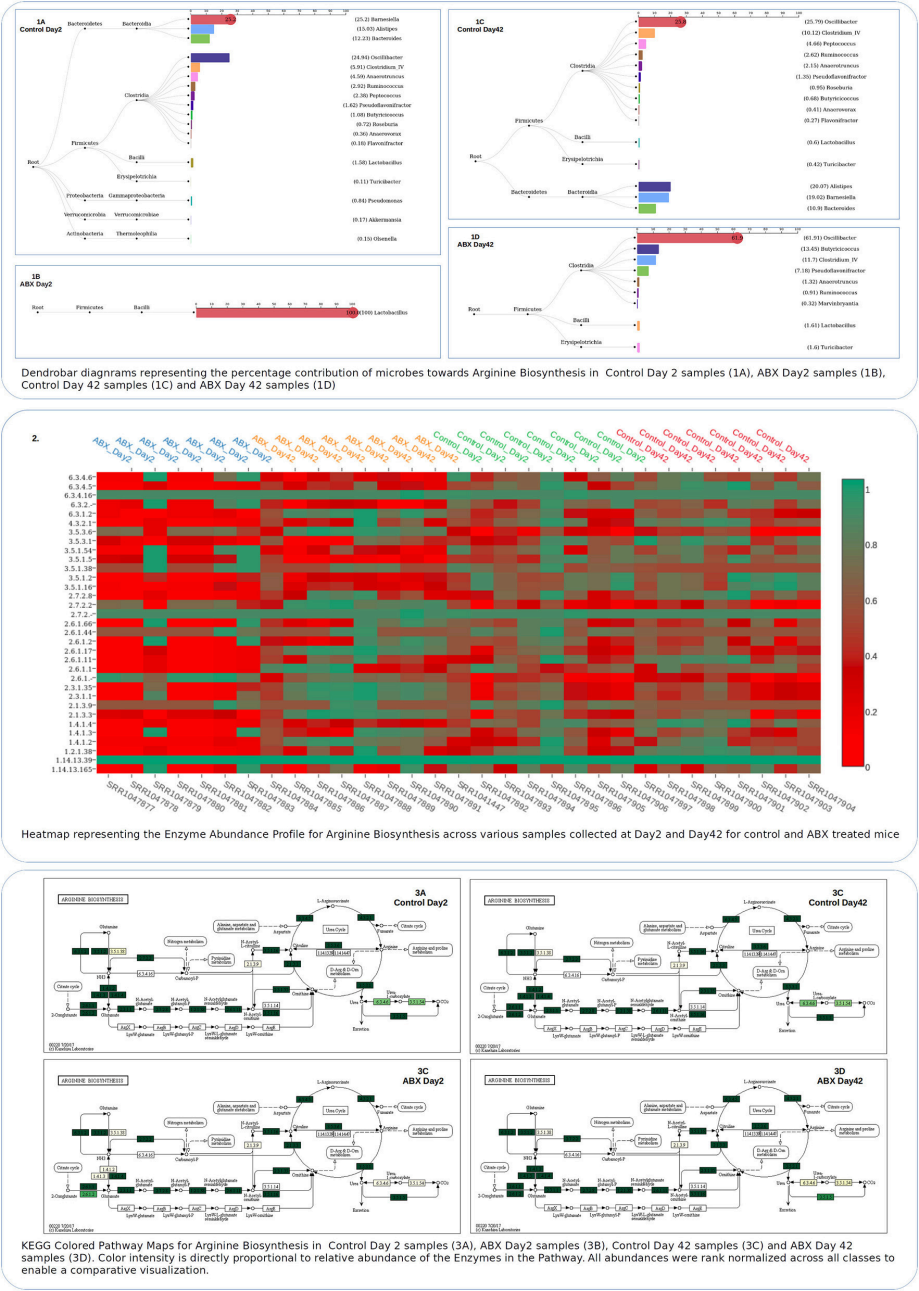
Results of iVikodak's Local Mapper Module for datasets corresponding to case study 1. Plots for (1) Contributors' dendrobar (2) Pathway specific enzyme profile (3) Colored KEGG Path way Map pertaining to Arginine Biosynthesis for case study 1, qenerated by Local Mapper.

Figures 2–**4** represent a graphical ensemble of (a subset) of key results generated by iVikodak for the aforementioned dataset. The array of results exemplifies the substantially expanded scope/breadth of functionalities available in iVikodak as compared to that in Burrito (McNally et al., 2018) for the same dataset (Supplementary File 1). The images in panels 1A and 1B of Figure 2 depict ordination (JSD-based PCoA) results grouped at two levels viz. as per nature of samples (controls vs. treated) and according to treatment time-points (Day 2 and Day 42 for both controls and treated). It may be noted that the ordination was performed using the functional profiles (of respective samples) that were inferred using Global Mapper module of iVikodak and were automatically (pre)processed for enabling the ordination functionality. While results in panel 1A display the expected trend of segregation between control and treated samples, the clustering profile in panel 1B exhibits a biologically relevant observation, wherein temporal segregation is limited only to the treated samples. Panels 2A and 2B (Figure 2) also enable end users to explore, concomitantly visualize, and download a single visual (a readymade box-plot) that captures the pattern of top abundant functions in the provided samples at two different levels of functional hierarchy. Furthermore, iVikodak provides to end users a combined (and easily customizable) heatmap depicting the “core” set of functions (which are “high” as well as “consistently” abundant) in provided sample (and sample classes). Visuals generated for this functionality (depicted in panel 3A and 3B of Figure 2) along with that depicted in panel 2 (for the analyzed case-study datasets) enables end users to easily comprehend the relative abundance pattern of various key functions in the analyzed samples.

The (function-driven) taxa correlation networks depicted in panels 4A–E of Figure 2 unravel quite a few interesting biological insights. These networks clearly depict a state of dysbiosis in gut microbial communities treated with antibiotics. Comparison of networks in panels 4A and 4B visually depicts a marked breakdown of functional interactions between various microbes constituting the gut microbiomes in treated states. It is interesting to note the complete absence of any functional correlations (between any of the members in the bacterial community) 2 days post-antibiotic treatment, and the re-appearance of some interactions 42 days post-treatment. Although this represents an interesting scientific observation (with respect to the immediate impact of antibiotic treatment) whether this represents a true biological event or is it a mere statistical artifact (owing to sample size) remains to be probed further. Overall, it may be noted that these interesting findings (with respect to functional correlation based taxonomic interaction patterns) wouldn't have been obtained using other available analogous tools in this field of research.

The set of images depicted in Figure 3 enable end users to view (in context) the specific set of functions that display a statistically significant difference in their abundance across the analyzed sample classes. It may be noted that the ISFA module of iVikodak facilitates automated multivariate differentiating feature analyses (both pair-wise as well as multi-class). Of note is ISFA's ability to generate and depict differentiating functions in form of a (three-level) cladogram (representing the functions at all three levels of hierarchy). From the perspective of the present case-study, the cladogram panel in Figure 3 indicates the relative and significant depletion of various functionalities in antibiotic treated samples. The interactive downloadable taxa-function contribution tables represent a value-add to users intending to further decipher specific functions and/ or taxa of interest.

Taking cues from the results of Global Mapper and ISFA with respect to the depletion of functions related to amino acid metabolism (in antibiotic treated sample class) and also the corresponding observations in both Burrito (McNally et al., 2018) and previous reports (Theriot et al., 2014), we set forth to employ the Local Mapper module of iVikodak to probe this aspect in further detail. For this purpose, we chose to investigate “Arginine biosynthesis” using the same as an example query. Results (depicted in form of intuitive dendrobars in panel 1A–D of Figure 4) provide end users a detailed visual insight with respect to the contribution of various microbes (in context of their taxonomic lineage) to this specific function across various classes of the analyzed data. It is apparent from the results (panels 1C and 1D in Figure 4) that while contribution of microbes toward this function gets depleted immediately post antibiotic treatment, it gets restored a few weeks post-withdrawal of antibiotic administration. The heat map depicted in panel 2 of Figure 4 represents the abundance profile of various enzymes constituting this particular function of interest. The heatmap pattern appears to be more or less in sync with previously stated observations. Unlike other existing analogous tools, iVikodak provides readily up-loadable (formatted) KEGG map files that enable end users to generate colored pathway maps visually indicating the difference in enzyme profiles intuitively in a format as depicted in panel 3 of Figure 4. The KEGG colored pathway map and heatmap depicting the enzyme profile of Arginine Biosynthesis further substantiate the differences between the samples from controls and antibiotic treated mice (as well as the differences at various time points, once again highlighting the extensive metadata handling capacity of iVikodak).

In contrast, the graphic layout depicted in Supplementary File 1 primarily represents the type of visual analysis that Burrito (as a tool) enables. As seen from this figure, the visualizations generated by Burrito are primarily confined to two broad categories – (a) an interactive grouped/ stacked bar chart that allows end users to visualize the types and proportions of various taxa and functionalities in each uploaded sample (and sample classes), and (b) a collapsible dendrogram that helps in understanding the identified/ predicted functions in their respective hierarchical context. Viewed in the context of the present case study, although the generated charts do provide users an overall sense of important taxonomic and functional differences between the uploaded sample classes, the visualizations remain limited to “only” two types. Hovering over these visualizations pops up information regarding name and proportions of taxa/functions in form of tool-tips. Downloads of visuals highlighting/ showcasing any or all of the displayed information is in form of screen-grabs/SVG's requiring significant post-processing efforts to make them amenable for scientific publication. Overall, even viewed at a mere level of comprehensively meeting e-enablement and automation requirements, a lot of questions, scope and/ or utilities still remain unaddressed by Burrito.

In summary, the results of the above case-study presented in Figures 2–4 represent the structured and logical connection between the three modules of iVikodak. Commencing from Global Mapper, end users can first probe the data at a community level and then proceed for a comparative analysis of various classes of interest using the next module i.e., ISFA. The deciphered functions of interest from both previous modules lay the perfect context for ultimately performing a detailed visual/ exploratory (and statistical) investigation using the Local Mapper module.

### Other Case Studies Highlighting the Functionalities of iVikodak

Five pre-executed jobs are provided as case studies to exemplify various functionalities of all three modules of iVikodak. Job IDs 50a7bef1a5, 998f4e89e5, and d819c619f7 represent “dashboards” for results of Global Mapper, ISFA and Local Mapper for the available Periodontitis microbiome datasets (Aas et al., 2005; Griffen et al., 2012; Souto et al., 2014; Kirst et al., 2015). Briefly, analysis indicates a distinct functional signature common to diseased samples (in contrast to individual-specific pattern in healthy samples). The common set of top core functions identified is in line with functions expected in an oral environment. Distinct changes are observed with respect to the contribution of microbes toward functions that significantly differ between healthy and diseased states. Similarly, Job IDs 5cb3a79c2a and 6c32ef5cda represent results for complex environments (Navarrete et al., 2015; Derycke et al., 2016) and human body sites (Cui et al., 2012; Griffen et al., 2012; Human Microbiome Project Consortium, 2012; Alekseyenko et al., 2013; Botero et al., 2014; Kato et al., 2014; Romero et al., 2014; Xiao et al., 2014) respectively. Details of case studies are provided in Supplementary File 2.

## Implementation

### Overview

IVikodak is a multi-modular framework that enables inference of functional capabilities of a given microbial community, as well as provides an array of automated analytical methods and visualization options for the inferred function data. The three modules in the platform are logically inter-connected to enable automated functional metagenomic analyses in a structured manner, thereby offering a standard working procedure for inferred function driven metagenomics. iVikodak is implemented as a php based web platform developed with modern design practices. The platform employs a job-id and personalized (yet non-intrusive) dashboard-driven task management framework that facilitates seamless end user access to all available utilities and modules without the need for registration and/or the step of sharing personal information (Figure 1).

### Input Requirements

All modules of iVikodak primarily require two input files (i) Multivariate taxonomic abundance data File (ii) Multi-column metadata of various samples in the taxonomic abundance data. Supplementary (Video) File 3 and Supplementary File 4 represent a video tutorial and a schematic representation (respectively) describing the format of a sample taxonomic input data and the corresponding metadata file. Appropriately placed video tutorials, documentation and sample files embedded in tool tips of various modules of the platform, also attempt to provide a succinct guide to the end-users. Supplementary File 5 contains a listing of various objectives or functions that iVikodak is enabled to provide and perform along with a description of steps (SOP) to be followed for achieving the intended results as well as visualizations.

It may be noted that the existing function inference/prediction tools infer functional abundances using taxonomic input information that have been generated by querying (*in silico*) 16S rRNA gene sequences against “specific reference databases.” For example, default implementations of Tax4Fun, Vikodak, and PICRUSt employ (i.e., use as input) taxonomic profiles generated by performing *in silico* comparisons of query sequences against reference taxonomies in SILVA (Pruesse et al., 2007), RDP (Cole et al., 2014), and Greengenes (DeSantis et al., 2006) databases, respectively. Absence of cross-compatibility between tools and the input formats they require therefore presents a challenge to end users. To address this, iVikodak has been enabled to auto-detect, (re-)format, and appropriately process input taxonomic profiles generated using any of the three mentioned reference databases/tools.

### Outputs

Each module of iVikodak generates a comprehensive ensemble of “Textual output files” (downloadable as RESULTS.zip from personal dashboards) and “interactive visualizations/graphs” corresponding to various analytical approaches followed in the module. Said output files/graphs are unique to functionalities of the module used, details pertaining to the same have been described in the following sections of the article.

## Methods, Modules and Functionalities

In order to establish a meaningful workflow for performing amplicon sequencing data based functional metagenomic analysis, iVikodak framework incorporates three logically inter-connected modules, namely: Global Mapper, Feature Analyzer (ISFA) and Local Mapper. An additional module, named “Recreator” is provided. The latter enables end users to re-create an entire dashboard using the “dashboard specific.dash file” retained by the user post completion of analysis (details in section “Web-front utilities and improvements”). A detailed description of each of three key modules of iVikodak is provided below.

### Global Mapper Module

This module enables users to computationally infer (and subsequently visualize and download) the functional potential of one or more microbial environments quantified in terms of the relative abundance of various metabolic pathways. These inferences are obtained by processing (user-supplied) input taxonomic profiles in a series of steps that involve-

- Enumerating the repertoire of genes/enzymes/proteins encoded by the set of microbes constituting the provided taxonomic profile
- Normalizing this information using 16S and functional gene copy-number information
- Cross-mapping this information to other functional units, and
- Estimating the relative abundance of various metabolic pathways (in terms of appropriate functional units)

A pre-compiled back-end database is employed by iVikodak's Global Mapper module for performing the first three steps. This database includes copy number and mapping information of over 2,900 enzymes, 15,500 proteins (Pfam: ~11,200; TIGRfam: ~4,300), ~4,600 COGs, and ~11,000 KOs corresponding to more than 33,000 prokaryotic genomes. This information was collated from IMG (Markowitz et al., 2012) and PATRIC (Wattam et al., 2014) databases. While the first three steps are completed at the back-end (in default automatic mode, as described in Vikodak Nagpal et al., 2016, the final estimation step in Global Mapper module requires end users to provide their preference regarding the functional assumption to proceed with. Choosing “Co-metabolism” (CoM) option results in computing the effective abundance of a metabolic pathway under the assumption that various microbes residing in an environment can pool together the functional units they encode and contribute to the overall functioning of that pathway (in that environment). The other option i.e., “Independent contributions” (ICo), assumes microbes in an environment to be independent functioning entities (with respect to the functional units they encode). Consequently, under this assumption, pathway abundances are independently computed for (and from) each individual microbe (resident in that environment) prior to obtaining their respective sums (which are considered as the effective abundances of respective metabolic pathways).

Besides providing various algorithmic options, the practical utility of the Global Mapper module lies in the variety of (enabled) functional insights that can possibly be queried, obtained, visualized, downloaded, and shared by end users. The option to upload metadata corresponding to each of the samples and directly overlay (and visualize each type of) metadata over the generated results (in an automated manner) is expected to vastly improve the overall visual-experience and aid in showcasing relevant functional insights and differences between environments. Overall, the e-enablement efforts put in behind this module facilitates end users to look beyond simple textual predictions/ inferences, and generate an array of interactive, customizable, publication-friendly graphics (coupled with metadata information of individual environments) in terms of the following functionalities –

- Top functions present in these environments (depicted as box plots). These functions may correspond to various pathways or modules or COG/Pfam/TIGRfam classes at various levels of functional hierarchy. Method for “Top Functions” computation is described in Supplementary File 6.
- Core functions that consistently have a minimum defined abundance in a given environment (depicted as heat-maps). Method for “Core Functions” computation is described in Supplementary File 6.
- Differentiating functions that exist between two or more environments (based on Wilcoxon-rank sum and Kruskal-Wallis tests) visualized in the context of a cladogram. Method for “Differentiating Functions” computation is described in Supplementary File 6.
- Ordination Analysis, wherein the inferred functional profiles of the environments under study are subjected to Jensen-Shannon Divergence (JSD) based Principal Coordinate Analysis (Arumugam et al., 2011), and visualized as 2D/3D interactive plots. The sample metadata (e.g., geography, disease status, age, sex, etc.) can also be overlaid on various data points (i.e., microbiome samples) in the generated plot. The latter overlay feature is applicable to all visuals generated by iVikodak.
- Function driven Correlation networks, wherein nodes represent microbes and an edge between two microbes indicates that they are (potentially) co-contributing to one or more specific functions. Various centrality measures (degree, betweenness, clustering coefficient, etc.), characterizing the generated networks, are also computed. It may be noted that the relative contribution of individual microbes (constituting a given environment) to an inferred functional unit is obtained by invoking the “independent contributions” algorithm (of Global Mapper) which assumes functional exclusiveness between members of the microbial community. Method used for generation of “Function-driven Correlation networks” is described in Supplementary File 6.

### Inter-sample Feature Analyzer (ISFA) Module

This module enables users to perform statistical (multi-class) comparison of functional profiles generated by the Global Mapper module. Wilcoxon rank-sum test and Kruskal-Wallis tests are used for statistical comparison of two or multi-class data, respectively. Both uncorrected as well as Benjamini-Hochberg (BH) corrected (Benjamini and Hochberg, 1995) *p*-values are reported for the features identified as having a significantly different abundance among the compared environments. Users are provided with two modes of operation, namely, rapid and batch mode. The “*rapid*” mode of operation enables (comparative) statistical analysis between functional abundances that have previously been inferred from taxonomic profiles corresponding to two or more environments. These abundances, preferably derived using iVikodak's Global Mapper module (may be generated using any other functional inference tool), are to be provided in typical multivariate data table format to the ISFA module.

The “batch” mode of ISFA, in contrast, works with “Zipped” input data (that is obtained as output from Global Mapper module). The zipped data, containing functional abundance profiles at various Pathway Exclusion Cut-off (PEC) thresholds (Nagpal et al., 2016), enables a consensus-driven differentiating function analysis. PEC threshold based filtering ensures that a pathway is reported as “present” and “functional” only when the (inferred) proportion of its constituent genes/enzymes exceeds a minimum quorum. Interactive cladograms and consensus heat-maps (indicating the list of differentiating functions across all PEC values) are also generated, when the ISFA module is operated in “batch” mode.

The highlight of the ISFA module lies in the “*interactive lists of differentiating functions”* that it automatically generates and displays in a “queryable” format. These lists in form of “filterable, sortable, and exportable” tables represent various inferred functions, bacteria contributing to these functions along with their corresponding quantum of contribution. Given that these lists are “queryable,” end users get the flexibility to probe, in real-time, the following two aspects –

- Any specific pathway (or pathway-class) of interest, so as to find the list of bacteria contributing to that pathway (along with a comparative view of the proportions in which these bacteria have contributed to various environments or sample classes), and
- Any specific bacterial taxon of interest, to estimate its contribution to various pathways (or pathway-classes) in one or more environments.

### Local Mapper Module

This module enables a granular-level analysis of user-specified pathway of interest. For a given pathway, end users can probe, visualize, and compare (between environments, and the samples they constitute), the inferred abundances of various enzymes constituting the said pathway. The customizable output, provided in form of a heatmap, in a way depicts the “functional coverage” of any selected pathway across samples or environments. For a given environment (or any other feature provided in metadata), the Local Mapper module also provides an advanced “dendrobar” output format that not only represents the contribution of individual bacteria to the chosen pathway, but also enables users to visualize these bacteria in the context of their taxonomic lineage. As in all other modules, end users are provided drop-down menu options to visualize samples grouped as per metadata. More importantly, iVikodak additionally provides KEGG (Kanehisa and Goto, 2000) color map and KEGG 3D map files corresponding to a user-specified pathway. The latter files are derived from normalized enzyme abundance profiles of the chosen pathway. These ready-to-use pre-formatted output files can be directly uploaded by end users to the KEGG Mapper module of www.genome.jp (KEGG web-server) to generate a colored pathway map and a 3D pathway map (for graphically visualizing relative enzyme abundances).

## Web-front utilities, UI and UX

In order to provide a seamless user-interface (UI) and user-experience (UX) through a highly interactive and responsive web application, iVikodak uses modern design practices and its front-end employs contemporary state-of-art technologies including bootstrap 3.0, D3.js (Bostock et al., 2011), plotly.js (Plotly Technologies Inc., 2015), cytoscape.js (Franz et al., 2016), in-house java-scripts, etc.

As mentioned earlier, a stand-out feature of iVikodak worth a mention includes incorporation of a personalized and portable “Dashboard” feature. Jobs submitted to iVikodak are tagged to unique “job-ids” that not only help end users to secure or track job status, but also enables them to retrieve, visualize, customize, save, and instantly share the generated results with scientific peers through a personalized “Dashboard.” The latter is downloadable as a “.dash” file, which when re-uploaded, seamlessly re-creates the entire dashboard (even post-job ID expiry) for the end user. The dashboard feature in iVikodak is relevant given the present-day trend of open-science and scientific data collaboration or sharing. iVikodak incorporates a “live” task tracking system that provides users a real-time view and access of the following –

- Live status of jobs: Indicating time elapsed, stage of execution i.e., in-progress/completion, etc.,
- Access to intermediate textual results (as an when they are completed) so that users need not wait until the entire dashboard is created.

In addition, post job-completion, the dashboard indicates the total time taken to complete the given job. Apart from enhancing user experience, this feature provides users details pertaining to performance statistics for various submitted jobs. It may be noted that the time taken for various jobs is a function of not only the size of taxonomic abundance data, but also the scale or the types of metadata provided. It takes approximately 2 min for dashboard generation when a taxonomic abundance data comprises of 500 taxonomic features computed for around 100 microbiome samples with a two-column metadata. It may be noted that the live-tracking system enables users to instantly access (within 10–20 s) the functional profiles that are inferred by iVikodak from the uploaded taxonomic data. It is pertinent to note that the described task management approach and associated feature of dashboard-driven personalization is prominently absent in tools analogous in functionality to iVikodak.

## Future development and enhancements

Following are the key enhancements planned for iVikodak –

1. **Algorithmic cross-compatibility**: Currently, functions in iVikodak are inferred using algorithms and methodologies as described in Vikodak (Nagpal et al., 2016). The resultant (inferred) functional information is suitably re-processed (at the backend) and subsequently provided to end users through an interactive visualization interface. As a future enhancement, iVikodak is planned to enable end users to upload functional information inferred using algorithms other than Vikodak e.g., PICRUSt (Langille et al., 2013) and Tax4Fun (Aßhauer et al., 2015). Such uploaded data will also be appropriately re-formatted for enabling respective analyses and visualizations.
2. **Functional Scope:** It is planned to incorporate KEGG modules (Kanehisa and Goto, 2000) and Gene Context based Modules (GCMs) (Bhatt et al., 2018) to the current scope of KO, COG, Pfam and TIGRfam inference.
3. **Custom phylogeny:** The present version of iVikodak accepts taxonomic input data generated using any of the three popular taxonomic classification frame-works (viz. RDP Classifier, Greengenes, and SILVA). Post processing, it currently employs (in its backend) a pre-built taxonomic hierarchy to display respective taxonomic cladograms in various outputs that it generates. The scope of this functionality is planned to be enhanced to enable end users to upload (along with input taxonomic profile data) customized taxonomic as well as functional hierarchies that they wish to use in respective downstream visualizations.
4. **Prediction of Microbe-Disease associations:** In order to derive clinically actionable insights from iVikodak's functional inferences, we intend to enhance functionalities in iVikodak that can appropriately mine disease-specific (inferred) function profiles and decipher in quantifiable terms the association of specific sets of bacteria with a particular disease state (i.e., function driven Human Microbe-Disease Associations, HMDA). A global ensemble of conventional as well as recently developed (validated) algorithms/classifiers like Random Forest (Breiman, 2001), Adaptive Boosting (Freund and Schapire, 1997; Peng et al., 2018), NGRHMDA (Huang et al., 2017), LRLSHMDA (Wang et al., 2017) and other graph theory based approaches (Chen et al., 2017) to extend the utility of microbial co-contribution networks generated by iVikodak, are planned to be included in ISFA module of iVikodak.
5. **ReFDash Database** (Repository of Functional Dashboards): iVikodak's “dashboards” represent a comprehensive ensemble of information, results and visualizations pertaining to the (inferred) functional profiles of one or multiple environments/populations representing one or more microbial communities. Given the fact that each dashboard is unique and can be accessed using personalized JOB IDs, it is possible to create a repository of well annotated dashboards, using public data, as well as through community collaborations.

Given the above context, we intend to create a database (named as ReFDash Repository) with the following objectives –

1. Host pre-generated functional dashboards created using taxonomic profiles (and available metadata) corresponding to various environments.
2. Enable end users to upload, deposit and share their dash-files (or dashboards generated by iVikodak) with other members in the scientific community. The underlying objective is to encourage microbiome researchers to help expand the repertoire of environments (represented by dash files in the ReFDash database). This would ease the process of scientific data collaboration, sharing or even a peer-review process.
3. A further objective is to create a framework that facilitates (automated) “on-demand” (reprocessing and) comparison of functional profiles of a subset of selected environments that are available as pre-generated/ end user deposited dash-files in the ReFDash repository.

Although, the above ideas are under active development, a prototype of the database may be accessed at https://web.rniapps.net/iVikodak/refdash/.

## Conclusions

iVikodak represents an effort to develop a one-stop “infer-analyse-compare-visualize” solution that can assist researchers in deciphering important biological insights with respect to the functional potential of microbial communities based on 16S rRNA gene sequencing datasets. The modular (yet inter-operable) framework of iVikodak intends to lay-down a standard workflow for inferred functional metagenomics. It can facilitate end users to concomitantly infer, statistically analyze, and compare multiple microbial communities, and in the process generate a plethora of intuitive self-explanatory visual outputs in an automated fashion. The ISFA and Local Mapper modules of iVikodak are logical extensions of the Global Mapper module and their expanded scope now enable end users to automate the statistical comparison between the functional potential of multiple environments (and their corresponding classes or metadata). The planned development (and eventual) linkage to ReFDash Repository represents the broad vision behind this work, and it is anticipated that iVikodak will add a significant value to the existing space of inferred function driven metagenomics space.

## Availability and Requirements

1. **Project name**: iVikodak
2. **Home page**: https://web.rniapps.net/iVikodak/
3. **Tutorials:** Each module has dedicated video tutorials. Comprehensive documented tutorials may be accessed at: https://web.rniapps.net/iVikodak/tutorials.documentation/
4. **Operating system(s):** Web platform compatible with all operating systems
5. **Browser requirement(s):** WebGl enabled for viewing 3D plots

For inquiries and general discussions, please contact sunil.nagpal@tcs.com, mm.haque@tcs.com or sharmila.mande@tcs.com.

## Availability of data and materials

Publicly available datasets were used in all the case studies described in this manuscript. A summary of their source(s) has been provided in Supplementary File 7. In addition, the taxonomic abundance (data) matrices, along-with their meta-data, for all the datasets used in this manuscript (in addition to other datasets) have been populated in the ReFDash database (https://web.rniapps.net/iVikodak/refdash/). Supplementary File 8 is an archive comprising of the mentioned datasets. This archive also includes (a) various scripts that enable regeneration of R plots from the output matrices generated by iVikodak, as well as (b) the code base for analytical work-flows for identification and generation of visualizations corresponding to top and core functions.

## Author Contributions

SN conceived, designed and developed the method, algorithm and web platform for iVikodak. SN and RS mined back end data and performed case studies. SN, MH, and SM conceived idea for ReFDash. SN, RS, and MH mined data for ReFDash. MH, SN, RS, and SM prepared the manuscript. All authors have reviewed and validated the platform and manuscript.

### Conflict of Interest Statement

The authors declare that the research was conducted in the absence of any commercial or financial relationships that could be construed as a potential conflict of interest.

## Abbreviations

WGS: Whole Genome Shotgun
PEC: Pathway Exclusion Cut-off
JSD: Jensen-Shannon Divergence
BH: Benjamini-Hochberg
ISFA: Inter Sample Feature Analyzer
PCoA: Principal Coordinate Analysis
ICo: Independent Contributions
CoM: Co-Metabolism
UI, UX, User Interface: User Experience.
