# Supplementary material for: iVikodak—A Platform and Standard Workflow for Inferring, Analyzing, Comparing, and Visualizing the Functional Potential of Microbial Communities
Source: Front Microbiol. 2019 Jan 14;9:3336. doi: 10.3389/fmicb.2018.03336 (PMC6339920; doi:10.3389/fmicb.2018.03336)

Supplementary file 1: Summary of results generated by Burrito web platform for temporal observation of differences in inferred function profile of gut microbiota of antibiotic treated mice.

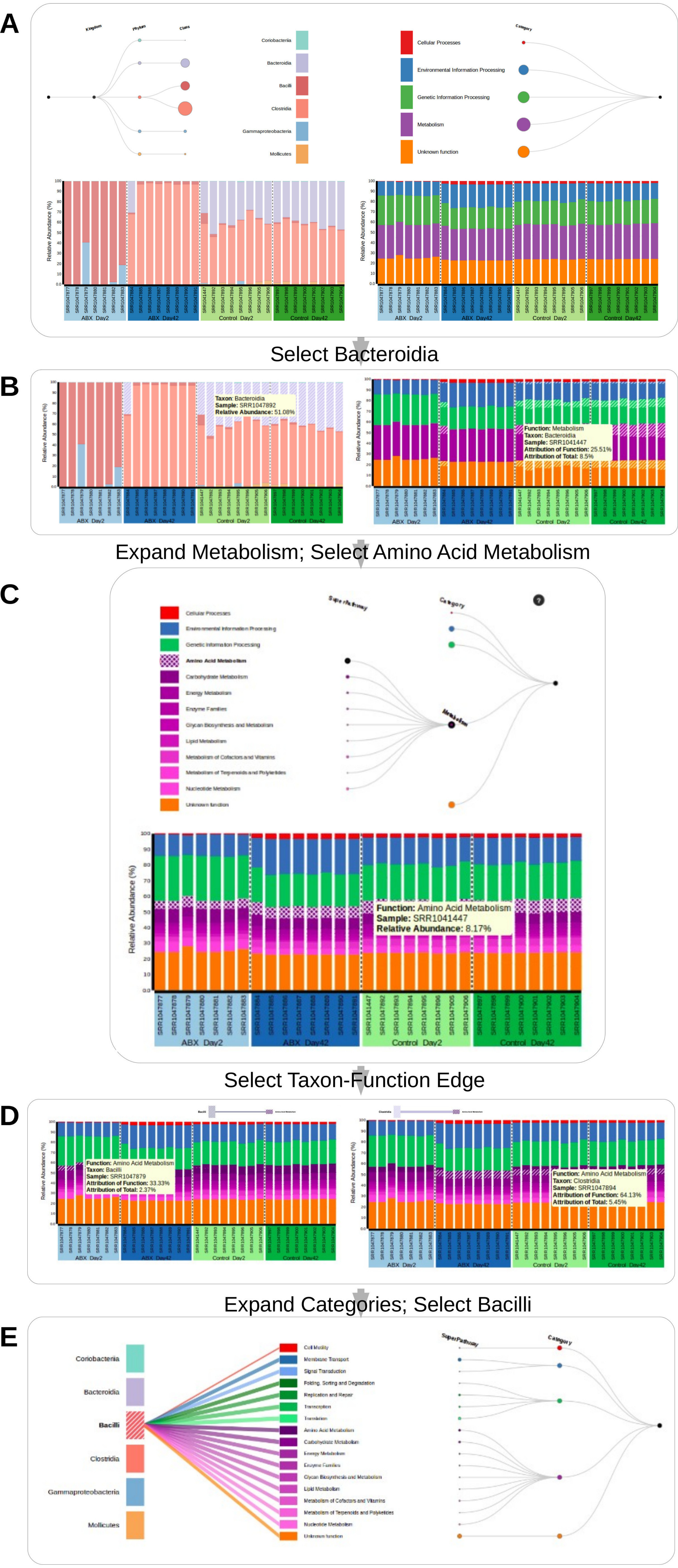

Supplement: Supplementary file 1 [file Data_Sheet_1.pdf]
