# Supplementary material for: iVikodak—A Platform and Standard Workflow for Inferring, Analyzing, Comparing, and Visualizing the Functional Potential of Microbial Communities
Source: Front Microbiol. 2019 Jan 14;9:3336. doi: 10.3389/fmicb.2018.03336 (PMC6339920; doi:10.3389/fmicb.2018.03336)
Supplement: Supplementary file 2 [file Data_Sheet_2.pdf]

Supplementary file 2: Detailed analysis report for case studies pertaining to Periodontitis, Human Body Sites and Complex environments is provided in this file

Index	
**Section/ Sub-Section**	**Access button**
Section 1: **Periodontitis Case Study (Data Summary)**	Click to go
Global Mapper - Ordination Results	Click to go
Global Mapper - Top Function Results	Click to go
Global Mapper - Core Function Results	Click to go
Global Mapper - Function Driven Networks	Click to go
ISFA - Differentiating Functions	Click to go
Local Mapper - Enzyme Profile	Click to go
Local Mapper - Dendrobars	Click to go
Local Mapper - KEGG 3D Map	Click to go
Section 2: **Human Body Sites (Data Summary)**	Click to go
Global Mapper - Ordination Results	Click to go
Global Mapper - Top Function Results	Click to go
Global Mapper - Core Function Results	Click to go
ISFA - Differentiating Functions	Click to go
Global Mapper - Function Driven Networks	Click to go
Section 3: **Complex Environments (Data Summary)**	Click to go
Global Mapper - Ordination Results	Click to go
Global Mapper - Top Function Results	Click to go
Global Mapper - Core Function Results	Click to go
ISFA - Differentiating Functions	Click to go
Global Mapper - Function Driven Networks	Click to go
Note: There is a button on Top/ Bottom of each page to bring the reader back to this index.	

Supplementary Section 1

Periodontitis Case Study

iVikodak was utilized for predicting as well as analyzing 91 publicly available 16S metagenomic datasets (Griffen et al., 2012) corresponding to sub-gingival microbiome samples from 30 Periodontally healthy control subjects (HC), and sub-gingival samples from 30 shallow (PS) and 29 deep (PD) periodontal pockets of subjects with Periodontitis. The objective of this case-study is to highlight, the biological relevance of iVikodak's functional predictions, and utilities of its visualizations.

Input data for the case study may be accessed “here” and the metadata for the same may be accessed “here”. These datasets were processed using all three modules of iVikodak and results were analyzed for functional significance. It may be noted that the subsequent sub-sections represent only 'graphical results and their corresponding observations/ arguments' for the chosen case study. These graphical visualizations are a mere sub-set of the whole repertoire of results (textual and graphical) that can potentially be generated by all the modules of iVikodak.

Results from Global Mapper

A. Ordination of Functional Profile(s)

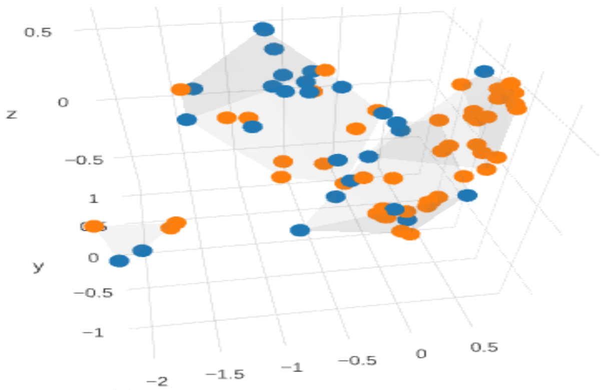

Fig S1 (a)

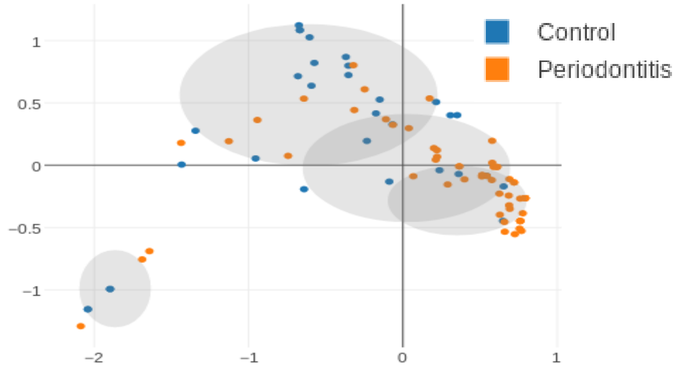

Fig S1 (b)

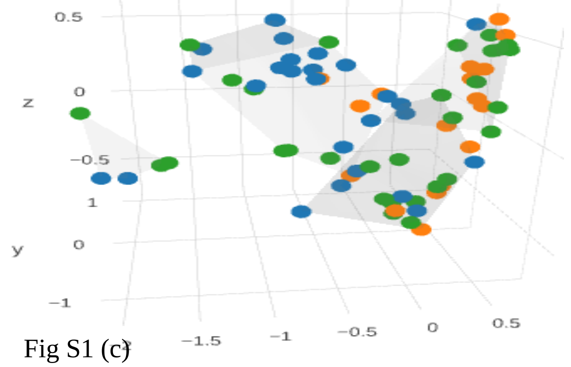

Fig S1 (c)

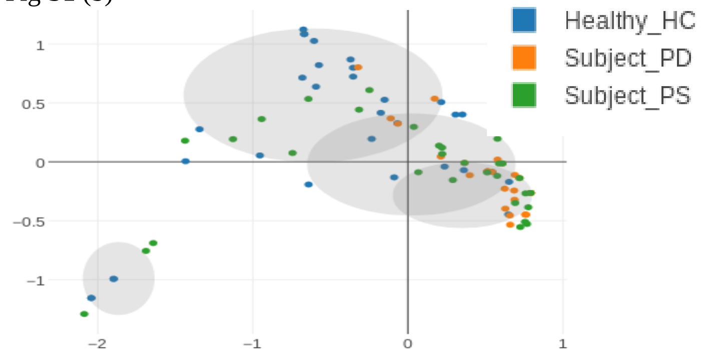

Fig S1 (d)

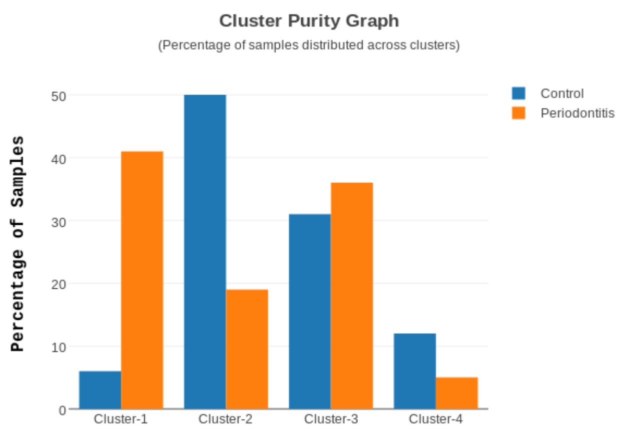

Fig S1 (e)

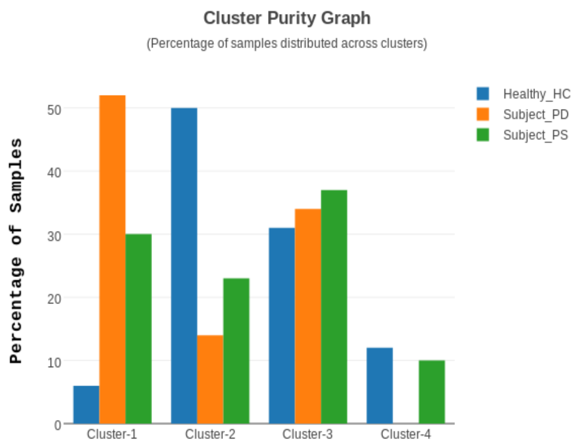

Fig S1 (f)

Figure(s) S1 *Results of (JSD based) ordination (PcoA) analysis automatically generated by Global Mapper module of iVikodak using the inferred function profiles for various classes of meta-data supplied for functional inference. Figure S1 (a) and (c) depict the 3D visualizations, while (b) and (d) represent the 2D visualization of Ordination results. Figures S1 (e) and (f) represent cluster purity graphs for both classes of meta-data (Nature: Periodontitis & Healthy; and Location: Periodontitis_PD (Deep pocket), Periodontitis_PS (Shallow pocket) & Healthy_HC (Healthy control). A cluster purity graph represents the proportions of samples distributed in various clusters of PCoA.*

Close clustering of samples pertaining to (Periodontitis) affected subjects could be observed, while samples pertaining to healthy controls were observed to have relatively spatially scattered pattern. The results hold biological significance given the fact that, in most cases, healthy subjects are expected to have microbial profile(s) that reflect individual-specific signatures (that reflect their individual state of periodontal 'health'). In contrast, presence of a common disorder/ diseased condition is expected to manifest itself into a relatively homogeneous signature of microbiome profile. The homogeneity could either be a cause or consequence of the disorder.

B. Top Functions: The results of Top (five) predicted functions generated by iVikodak at the specific level (L3) and at broader (L2) level of functional hierarchy are shown in Figure S2. The results indicate higher abundances of certain specific functions in both Peridodontitis and Control subjects. These include Purine metabolism, Pyrimidine metabolism, Amino sugar and nucleotide sugar metabolism, Glycolysis & Gluconeogenesis and Aminoacyl t-rna biosynthesis. A common pool of top functions in both affected and healthy subjects rather indicate the presence of a core 'house-keeping set' of functions that are common to the oral environment. Similarly, at level 2, Carbohydrate metabolism, Amino acid metabolism, Replication and repair, Metabolism of co-factors and vitamins, and Nucleotide metabolism constituted the top (five) functions.

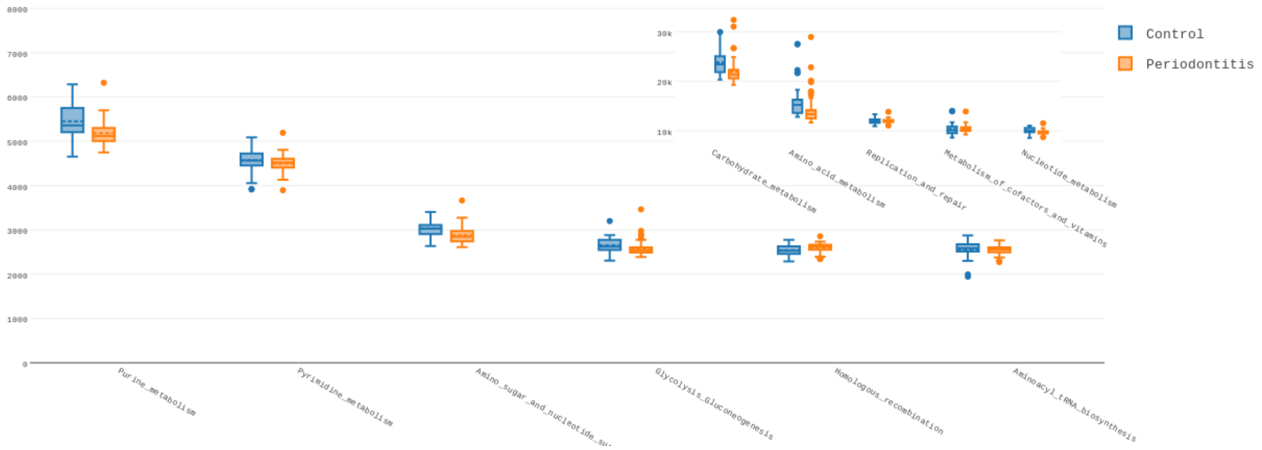

Fig S2: *Box plots depicting the Top predicted functions in Periodontitis and Control samples. The plot is automatically generated by Global Mapper module of iVikodak.*

Similarly, box plots for the other set of metadata (pertaining to the loci of sampling) were also generated.

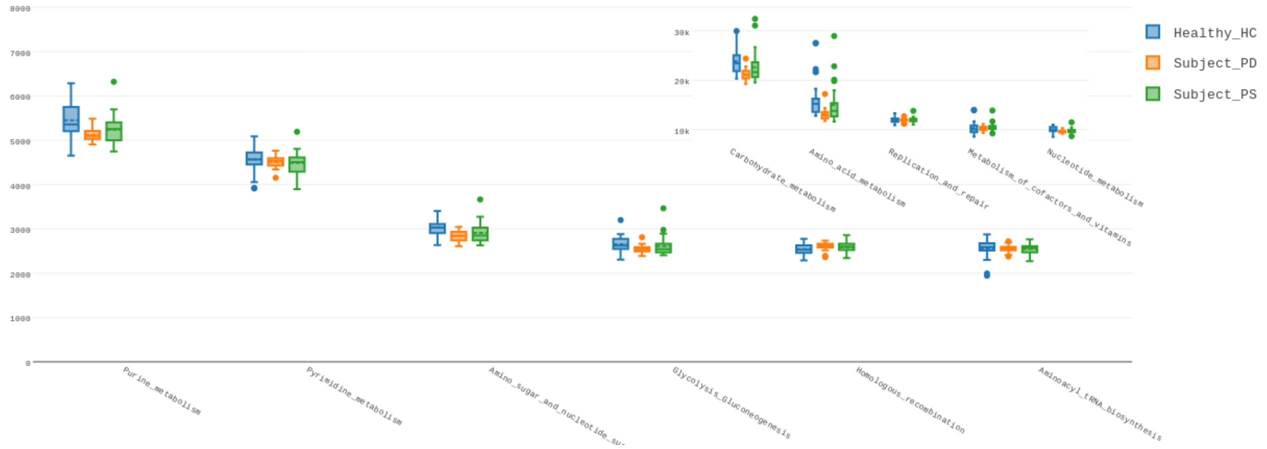

Fig S3: *Box plots depicting the Top predicted functions in Healthy (HC), Periodontitis_Shallow pocket (PS) and Periodontitis_Deep pocket (PD) samples. The plot is automatically generated by Global Mapper module of iVikodak.*

C. Core Functions: Global mapper module of iVikodak also infers the Core functions for the environments (pertaining to supplied metadata). A function is defined as 'core' if it is consistently abundant in at least 90% of the samples of a given environment with a median abundance of at least 20% of the most abundant function. Core functions are deduced through an iterative process, so that each core function gets a bootstrap score as an index of the confidence of deduction, and only those functions are visualized as core that cross the threshold score of 95 (i.e identified as core in 95% of the iterations).

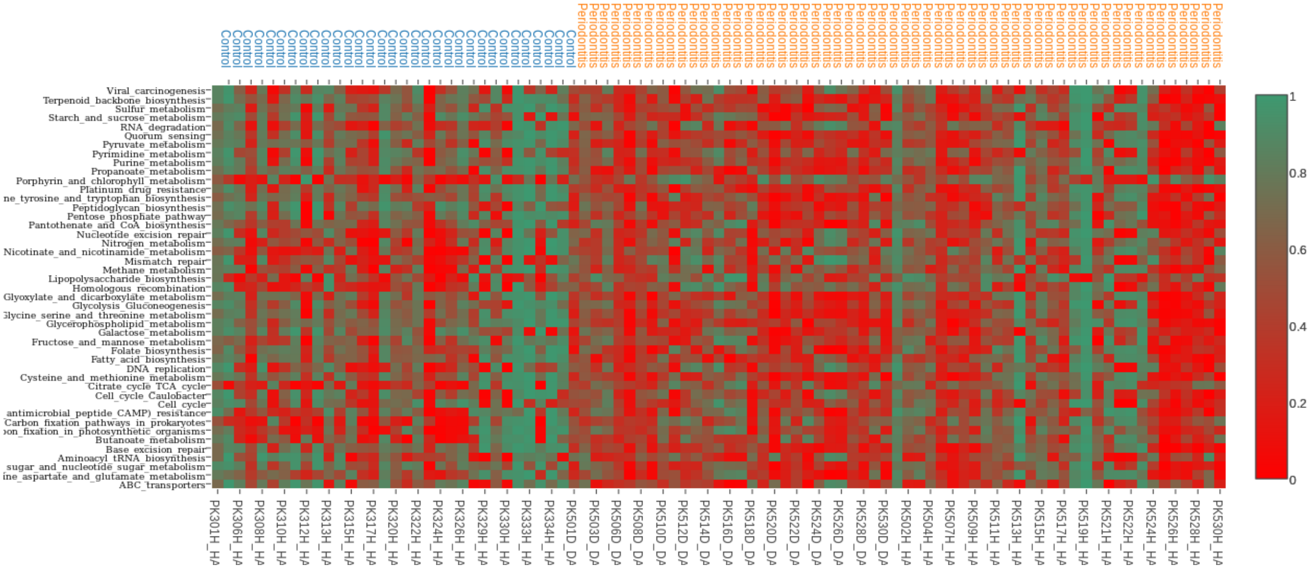

Figure S4: *Predicted Core functions for the microbial communities in the environments pertaining to Periodontitis affected and control subjects.*

The heatmap of rank normalized abundance profiles indicates an apparent difference in the abundance profiles of the predicted core functional pathways of the two environments. Core functions between Periodontitis and Control subjects were predicted to be more enriched in Control subjects as compared to the subjects with Periodontitis. The observation pertaining to the common core for Control and Periodontitis samples, holds biological significance as functions like pyrimidine metabolism, purine metabolism, starch and sucrose metabolism, quorum sensing, lipopolysaccharide biosynthesis etc., reflect the house keeping requirements of microbiota in the subgingival habitat. In addition, it is apparent that a substantial set of core functions get disrupted during transition from Control samples to Periodontitis affected samples.

Similarly, for the sampling loci based subgingival environment(s), the following core function profile was generated by iVikodak. Given that the computation of core functions is individually performed for each sub-class in the metadata, a different (and smaller) set of core functions was observed for the loci based subgingival environments. The common core though remained the same for loci based sampling as well (indicating the stable house keeping set of core functions).

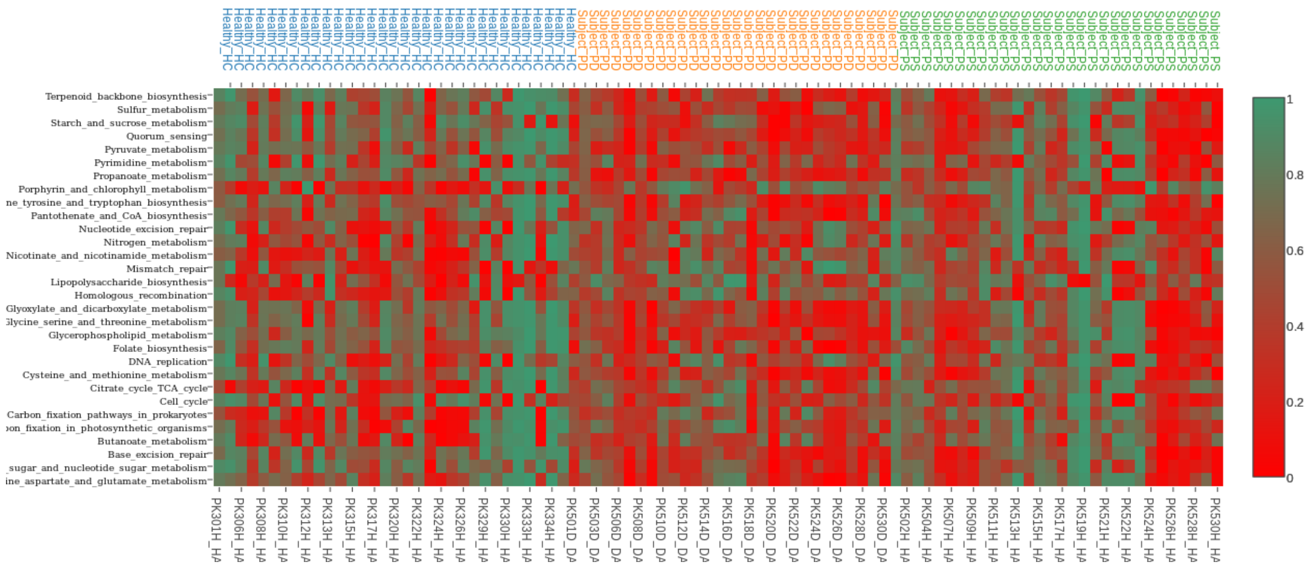

Figure S5: *Predicted Core functions for the environments pertaining to Periodontitis deep pocket site (PD), Periodontitis shallow pocket site (PS) and subgingival samples from control subjects (HC).*

D. Network Analysis: Vikodak introduced a novel concept of inferring inter-microbial interaction patterns using the correlations between their functional potentials (*Nagpal et al.*, 2016). The networks so obtained using such functional correlations were termed as 'Co-contribution' networks. Unlike co-occurrence networks derived from the abundance profiles of microbes in an environment, co-contribution networks (as introduced in Vikodak) were derived from functional contribution profiles of resident microbes of an environment towards various inferred functions.

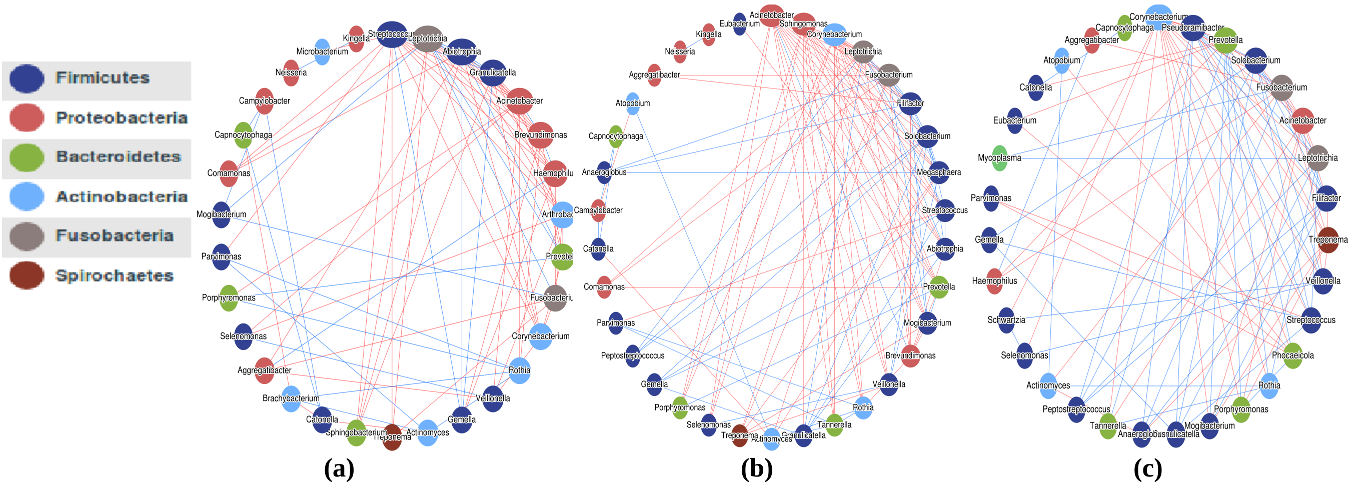

Figure S6 *Predicted function driven microbial interaction patterns in (a) Control environment, (b) Subgingival shallow pocket sites in Periodontitis subjects and (c) Subgingival deep pocket sites in Periodontitis subjects. Color of nodes represents the phylum affiliations of the microbes composing the network(s). A degree sorted circular layout has been used to visualize the network(s) which were automatically generated by iVikodak's Global Mapper module.*

The highest degree node in Control environment pertained to *Streptococcus* genus, which has been well reported as a common genus present in oral environment, and a member of phylum Firmicutes, which is also one of the most prevalent phylum in oral cavity. Streptococcus holds positive interactions (indicated by blue edges, as compared to negative interactions indicated by red edges) with *Granulicatella*, *Gemella* and *Abiotrophia* (all of which are members of Firmicutes phylum and predominant genera reported in oral environment) (*Aas et al*., 2005).

Upon transition to the network profile of subgingival shallow pocket sites of periodontitis subjects, an apparent increase in network membership is observed. The top degree nodes are constituted by bacteria belonging to Proteobacteria phylum as opposed to Firmicutes (the top degree phylum in Control subjects). *Acinetobacter* and *Sphingomonas* constitute the top degree genera for periodontal shallow pocket sites, both of which have been extensively reported to have pathogenic species for (human) oral environment (*Souto et al*., 2014).

The network profile for subgingival deep pocket sites of periodontitis subjects exhibited *Corynebacterium* (a member of Actinobacteria phylum) as the top degree node. The network membership was comparable to that of shallow pocket sites, but more than that of control environment. The next two genera amongst top degree nodes were *Acinetobacter* (from Proteobacteria) and *Pseudoramibacter* (from Firmicutes). Interestingly, all the interactions held by *Corynebacterium* with other members of the network were 'were observed to be negative'. Given that almost all the members of the network with which *Corynebacterium* interacts, belong to Firmicutes phylum (representative of control environment), a possibility of (opportunistic) pathogenic nature of the specific microbe pertainting to *Corynebacterium* genus is suggested here.

Inter Sample Feature Analyzer (ISFA)

ISFA module of iVikodak was used for comparative analysis of inferred function data obtained from Global Mapper module of iVikodak. It may be noted that, for multiclass comparison, iVikodak employs Kruskal-Wallis test, and Wilcoxon rank sum test is used for pair-wise comparisons. All p-values are corrected using BH correction.

Following are the graphical results obtained using ISFA module of iVikodak:

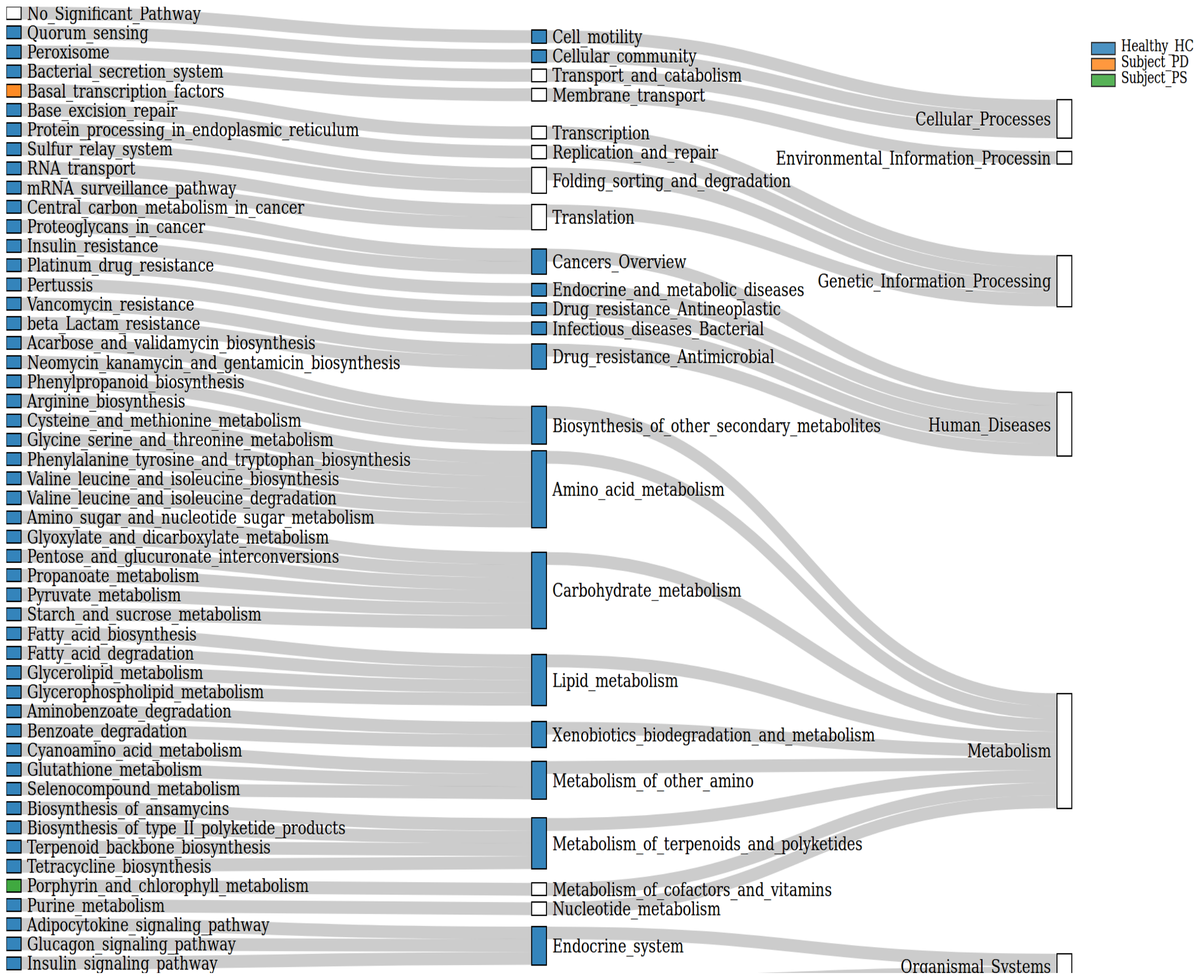

Figure S7: *(a) A cladogram of (Top 50 most abundant) significantly differentiating predicted functions between various subclasses of metagenomic environments pertaining to Periodontitis case study. Color of the nodes represent the class of samples in which the given function exhibits highest median abundance. White color signifies a non-differentiating function. The cladogram was generated using the raw functional abundance profile subjected to Kruskal-Wallis test by ISFA module, at a p-value cut-off of 0.001 (BH corrected).*

Predicted functions pertaining to Xenobiotics biodegradation and metabolism, Amino acid metabolism, Metabolism of co-factors and vitamins appeared significantly differentiating at all PEC values. The findings were consistent with previously reported observations (*Kirst et al*., 2015).

Local Mapper

Results of Global Mapper and ISFA modules of iVikodak predicted the significance of Purine metabolism (Core as well as significantly differentiating function) in the context of Periodontitis, amongst other important functional inferences obtained through iVikodak. Local Mapper module of iVikodak was therefore employed to deeply probe into this pathway.

Local Mapper generated a heatmap of enzyme abundance profile for Purine metabolism in the entire population of samples, and individual dendrobar diagrams, for each class of samples, representing the relative contributions of various microbes towards Purine metabolism. In addition, the 3D mapper files generated by Local Mapper were used to visually probe the relative abundance of various enzymes in various classes of samples.

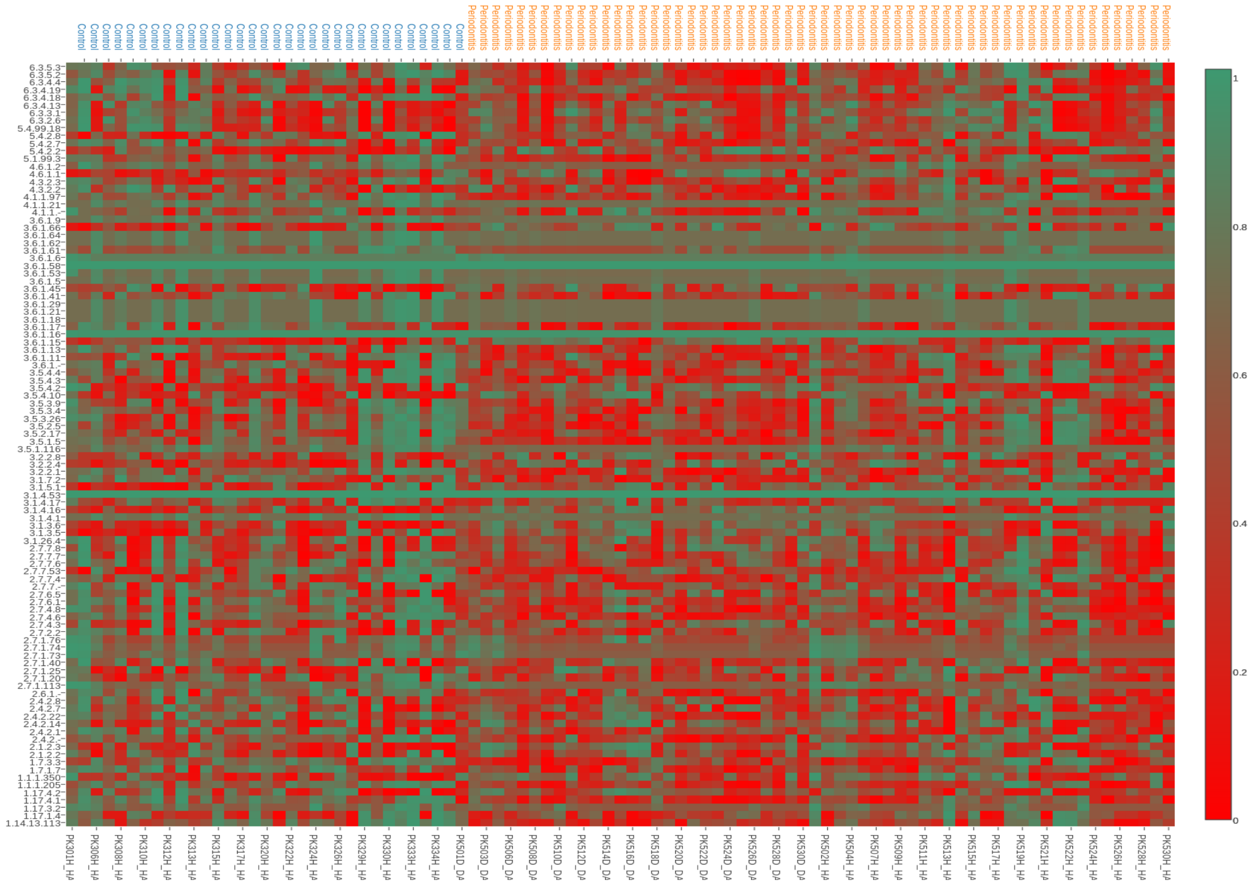

Figure S8: *Predicted enzyme abundance profile of Purine metabolism pathway across various samples of Periodontitis dataset.*

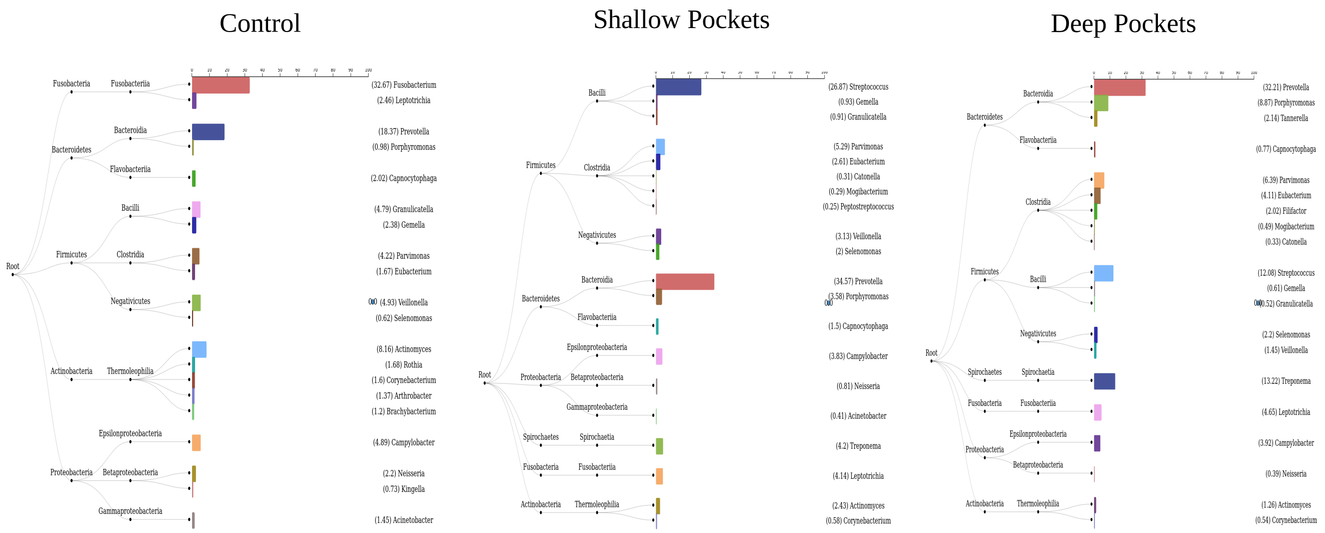

Figure S9: *Contribution towards predicted Purine metabolism by various microbes profiled in samples pertaining to Control, Shallow pockets and Deep pockets. Fusobacterium and Prevotella consititute the major contributors towards Purine metabolism in Control samples, whereas Prevotella and Streptococcus contribute the most in Shallow pocket sites of subjects. In deep pocket sites of subjects Prevotella, Streptococcus and Treponema contribute the most towards Purine metabolism.*

The observation indicates an apparent housekeeping role of *Prevotella* in oral environment (in context of Purine metabolism) and a potential correlation of *Streptococcus* and *Treponema* genera with the progression of Periodontitis.

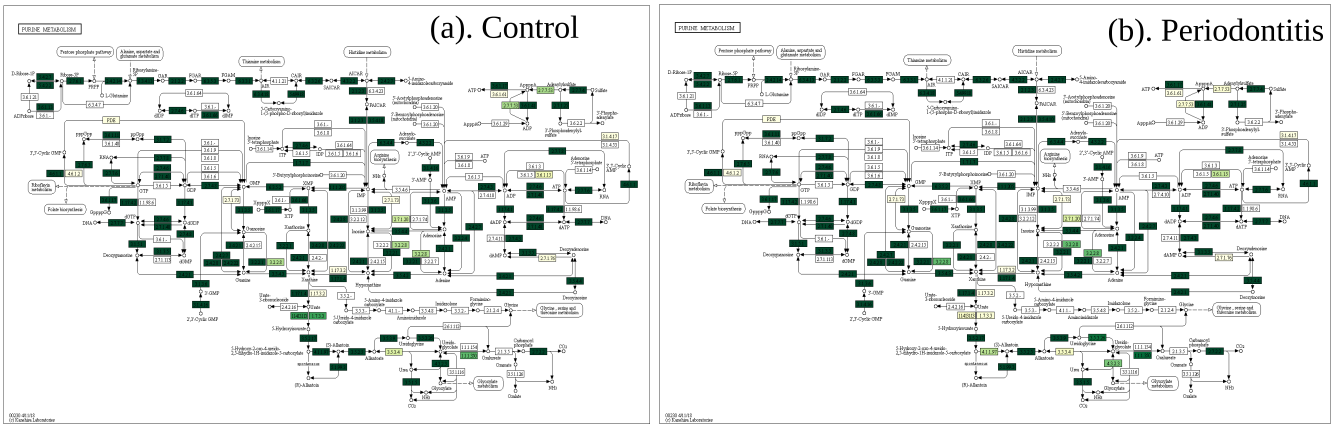

Figure S10: *Predicted enzyme profile for Purine metabolism pathway in Control and Periodontitis samples. The graphs were generated through KEGG Color mapper tool available at: http://www.genome.jp/kegg/tool/map_pathway3.html using the Color Mapping files generated by Local Mapper module of iVikodak.*

The profile suggested an apparent change in abundance profiles of enzymes involved in ATP synthesis, Allontoin synthesis, Xanthosine synthesis, and Oxalureate synthesis during transition from control to disease state.

3D map files were also used to generate the 3D bar embedded pathway visualizations for Purine metabolism on KEGG color mapper platform for individual enzyme specific visual comparisons.

(a). Control

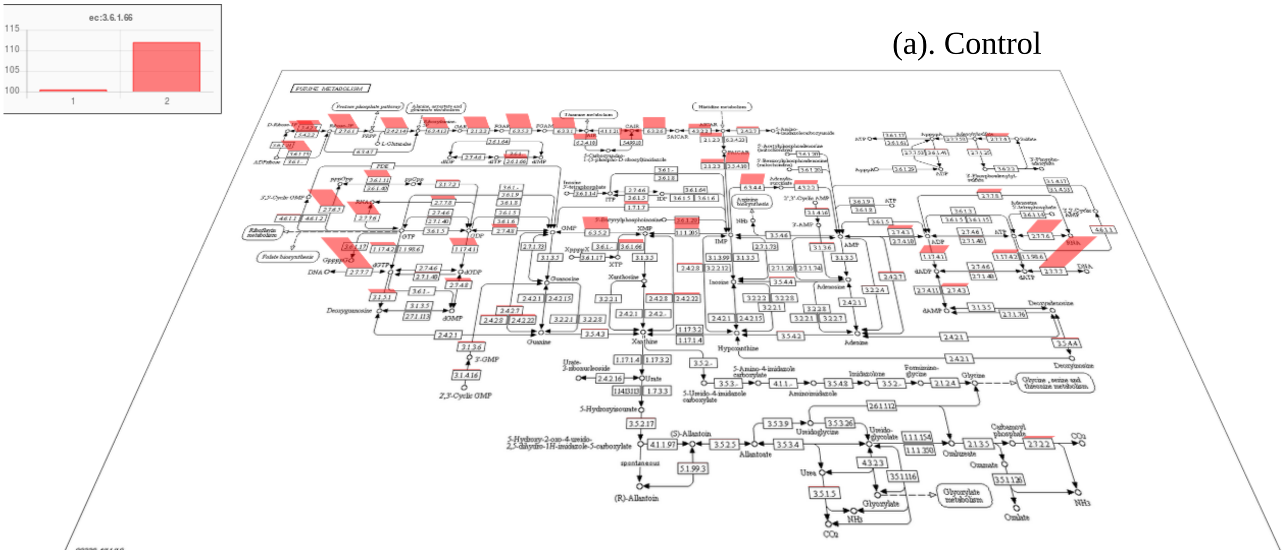

(b). Periodontitis

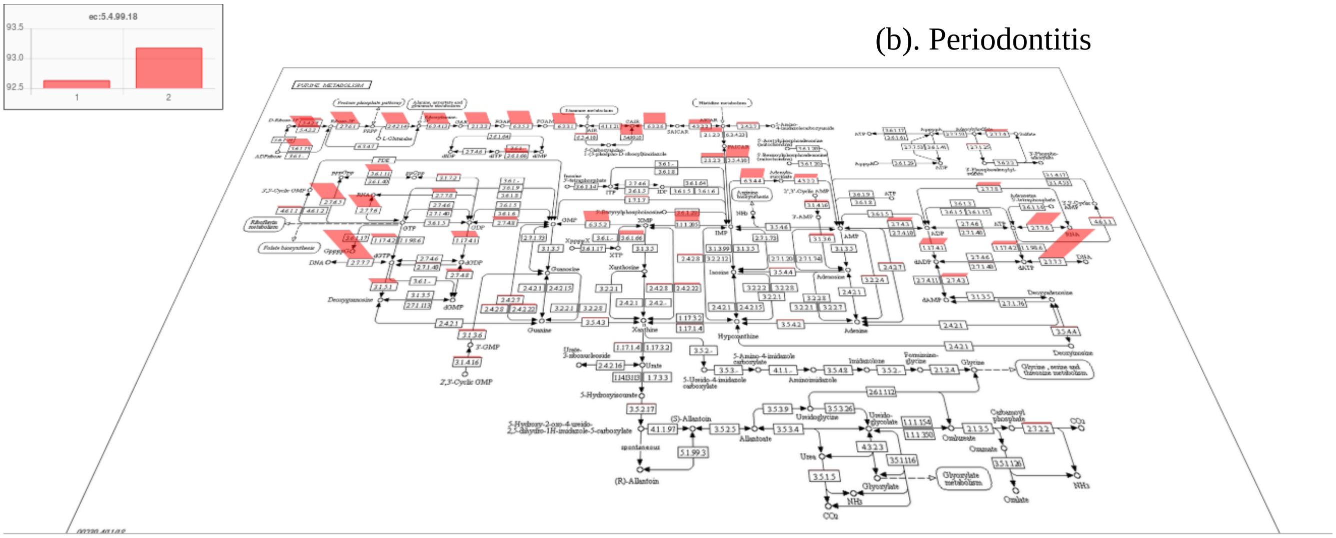

Figure S11: *3D bar embedded predicted enzyme profile for Purine metabolism pathway in Control and Periodontitis samples. The graphs were generated through KEGG Color mapper tool available at:* [*http://www.genome.jp/kegg/tool/map_pathway3.html*](http://www.genome.jp/kegg/tool/map_pathway3.html) *using the 3D Color Mapping files generated by Local Mapper module of iVikodak.*

Supplementary Section 2

Functional inference and analysis for 'Human Body Sites'

iVikodak was used to analyze a total of 1383 metagenomic samples from various body sites of human body. Table T1 gives a summary of the source of samples used in this case study:

Sample ID	Site	Geography	Datasets	Reference
Gut HMP	Gut (Human)	USA	306	Consortium THMP, 2012
Gut Prebiotics	Gut (Human)	Japan, China	283	Kato et al., 2014<br>Xiao et al., 2014
American Sputum, Oropharynx	Oral Cavity (Human)	USA	18	Botero et al., 2014
Chinese Sputum	Oral Cavity (Human)	China	55	Cui et al., 2012
Skin	Skin (Human)	USA	236	Alekseyenko et al., 2013
Sub-gingival	Oral Cavity (Human)	USA	91	Griffen et al., 2012
Vaginal	Vagina (Human)	USA	394	Romero et al., 2014

Table T1: Summary of datasets used for the Human Body Sites case study of iVikodak

Following are some of the key results and visualizations for this case study:

JSD based (automated) PCoA performed by Global Mapper module indicated distinct functional profile for various body sites. While samples pertaining to different body sites exhibited distinct spatial segregation, samples pertaining to physiologically similar body sites were observed to cluster together irrespective of the geography of samples. Figure SS1 represents the results of ordination of samples

pertaining to different (human) body sites using the inferred function profile derived from Global Mapper module:

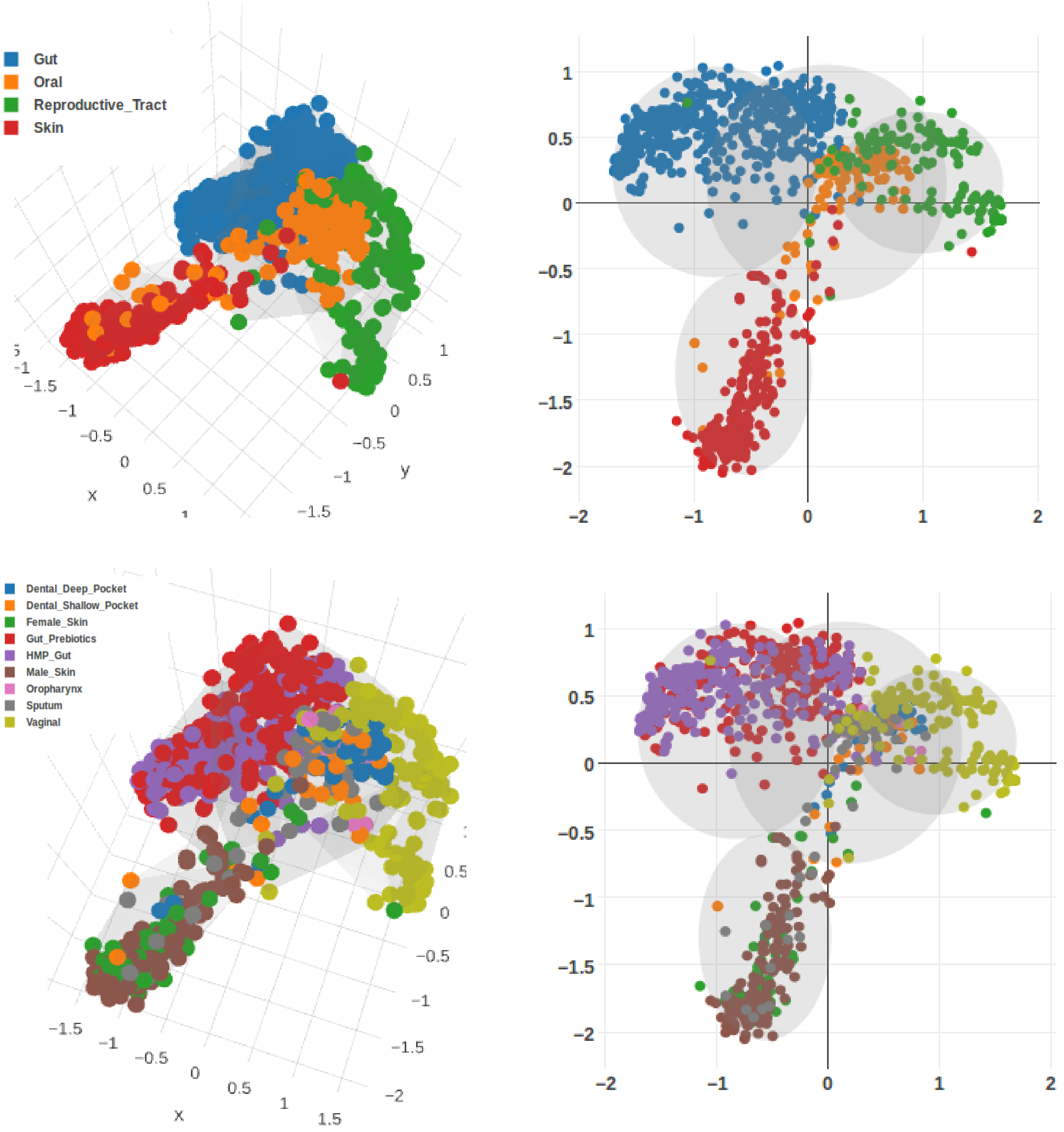

Figure SS1: Result of Ordination analysis performed on inferred function profile of samples pertaining to different body sites.

The cluster purity graphs (Figure SS2) further illustrate the findings of ordination analysis.

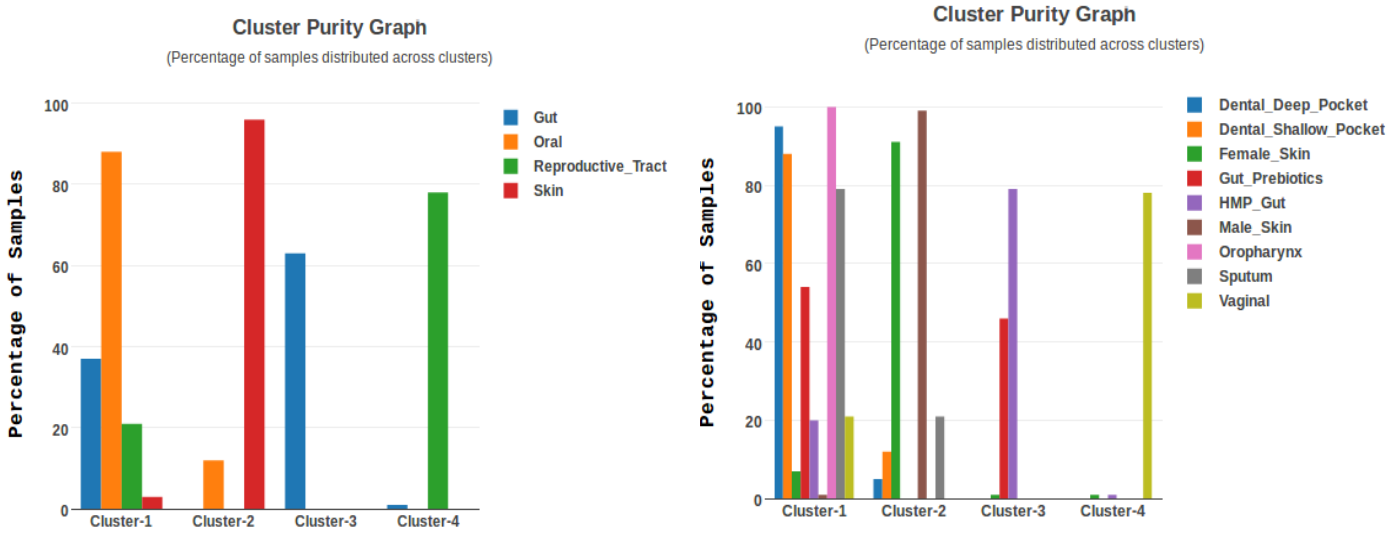

Figure SS2: Cluster purity graphs representing the proportional distribution of samples across various clusters during JSD based PcoA

A union of Top (five) functions for each of the body sites indicated an apparent and expected difference in the quantum of abundance of each of the Top functions acorss various body sites (Figure SS3).

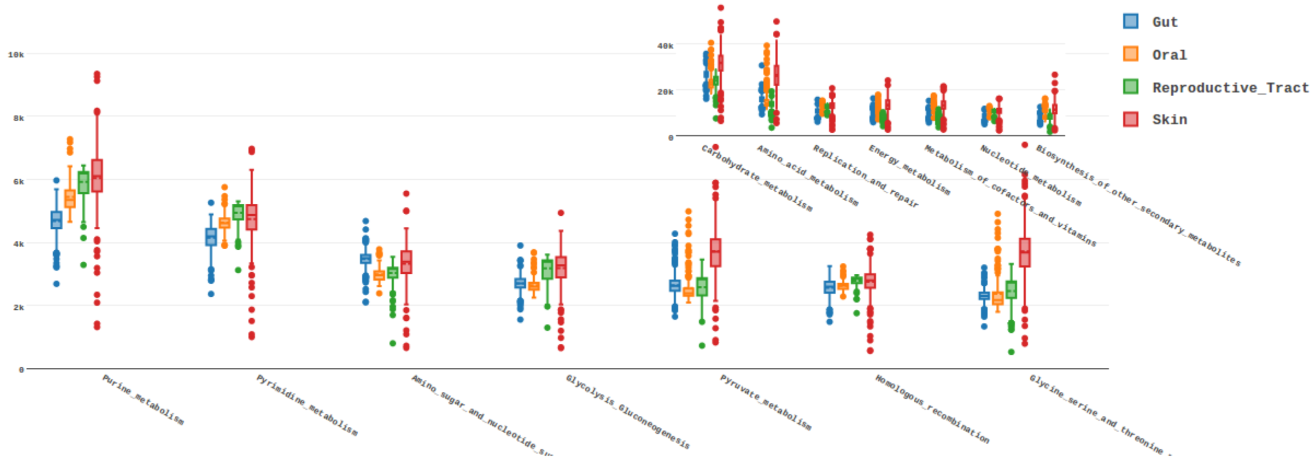

Figure SS3: Box plots representing the union of Top (five) inferred functions in each of the body sites

Analysis of core functions (as described earlier in section 1), indicated a distinct core function profile for each of the body sites (heatmap of the abundance profile of core functions clearly depicts the same in Figure SS4).

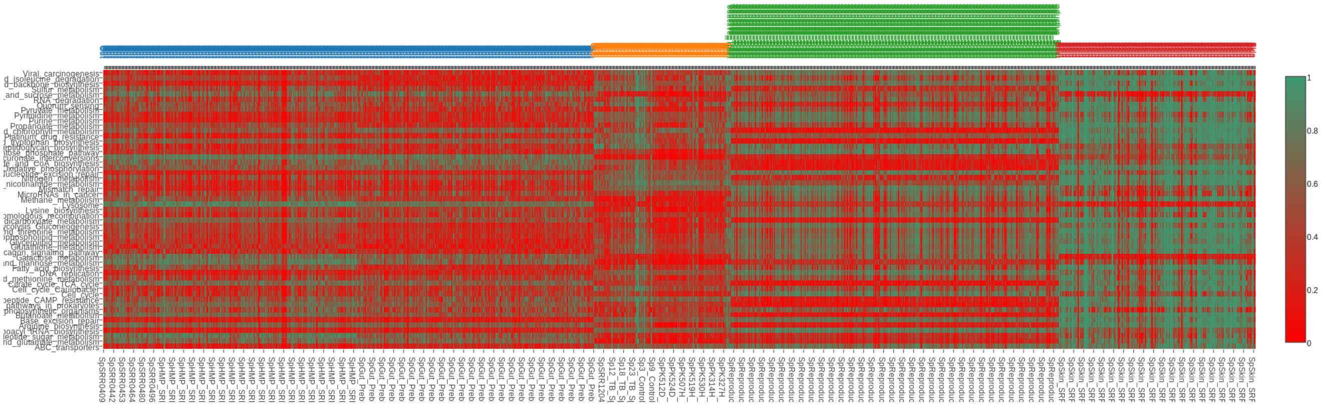

Figure SS4: Heatmap representing the (rank normalized) abundance profile of core functions across various body sites

Given the physiological distinction of various body sites, it was expected to have a diverse set of differentiating functions across different body sites. The cladogram in Figure SS5 represents the results of the differentiating function analysis across the body sites:

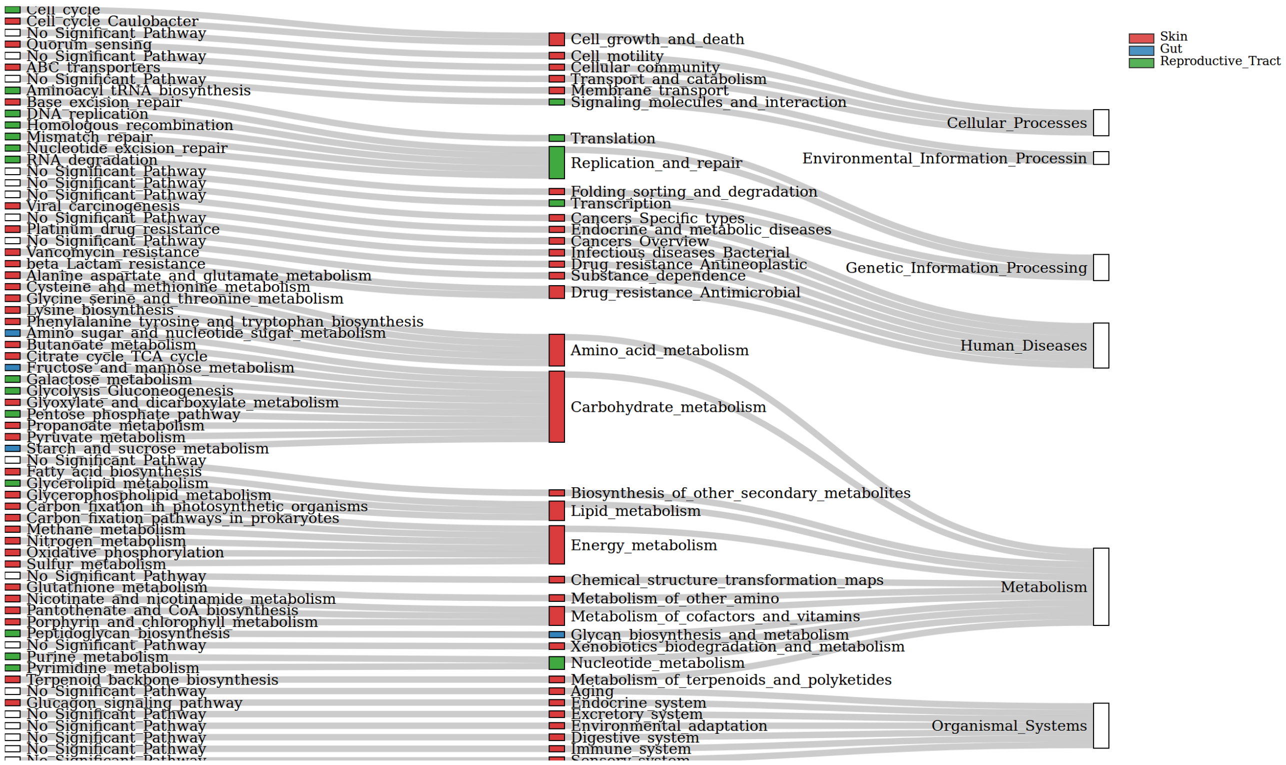

Figure SS5: Cladogram representing the differentiating functions across various body sites. Color of the node represents the body site in which the given function is significantly more abundant than other body sites.

In addition, function driven microbial interaction networks were also deciphered by Global Mapper module of iVikodak. Following are some of the network graphs for various body sites (Figure SS6):

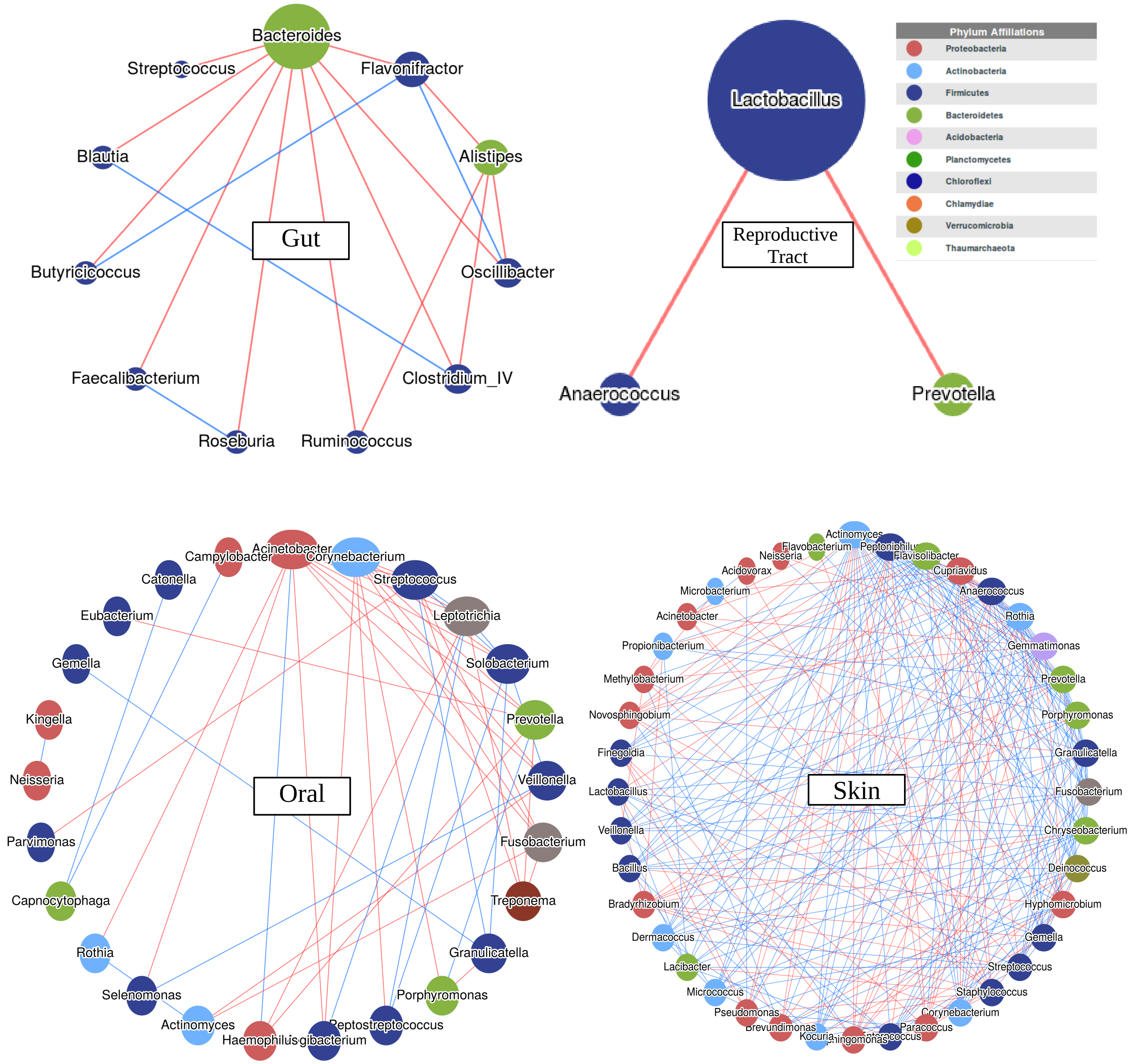

Figure SS6: Function driven microbial interaction networks for various body sites. Color of the nodes represent their phylum affiliations. Networks are displayed in degree sorted circular layout.

Similar analysis was performed on Complex metagenomic environments pertaining to Soil and Nematode metagenomes (DDBJ ID: ERA411828 and DDBJ ID: SRP064694 respectively). A summary of the key results and visualizations for the complex environments case study is provided below:

Supplementary Section 3

Functional inference and analysis for 'Complex Environments' (DDBJ ID: ERA411828 and SRP064694).

A. Ordination

Automated JSD based PCoA for inferred function profiles of Nematode and Soil metagenomic samples was performed by Global Mapper module of iVikodak.

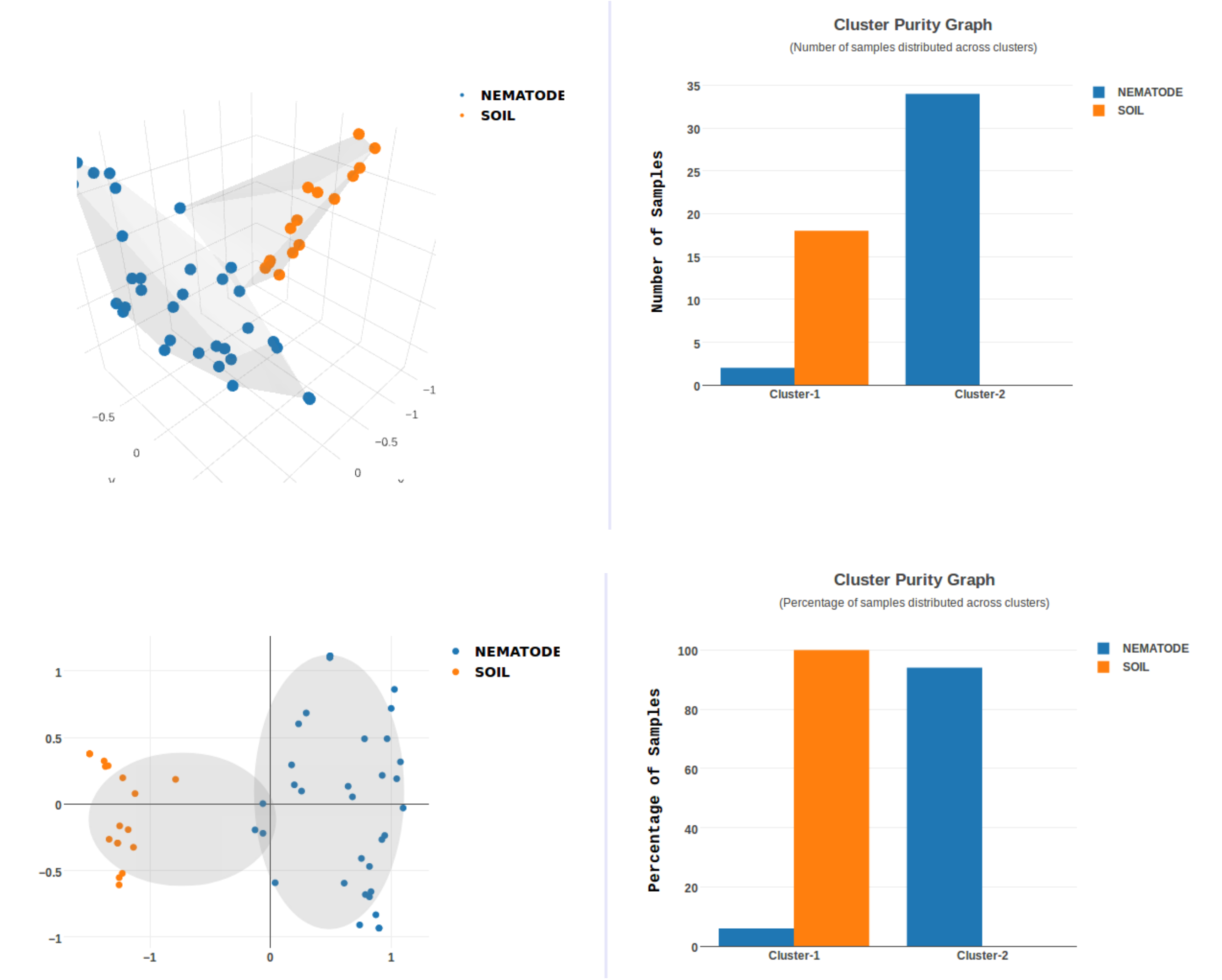

Figure SS7: Results of function driven ordination analysis (JSD based PCoA) for Complex metagenomic environments (i.e Nematode and Soil) using the Global Mapper module of iVikodak

B. Top Functions

An apparent difference was observed in the abundances of Top Functions for Nematode and Soil metagenomic environments.

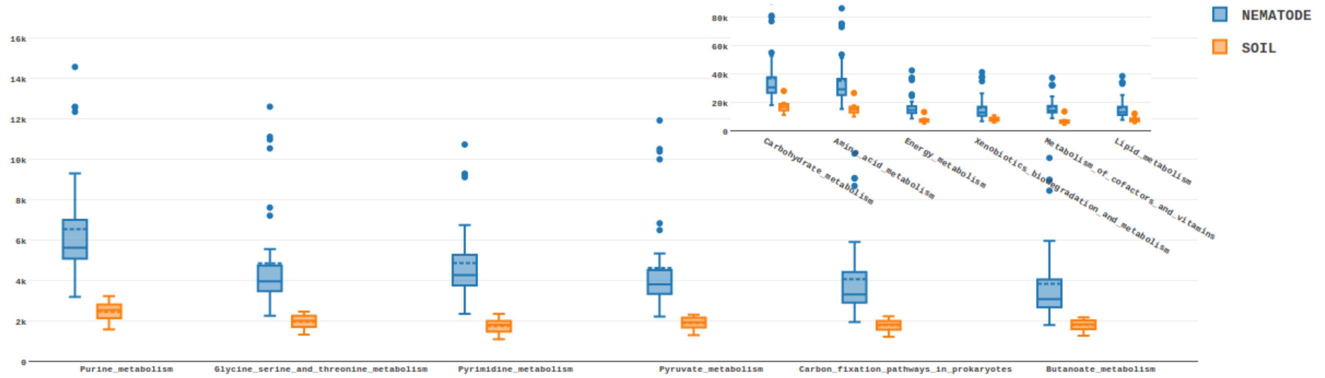

Figure SS8: Box plots depicting the Top functions for Nematode and Soil metagenomic environments

C. Core Functions

Both Nematode and Soil metagenomic environments exhibited a very distinct core function profile:

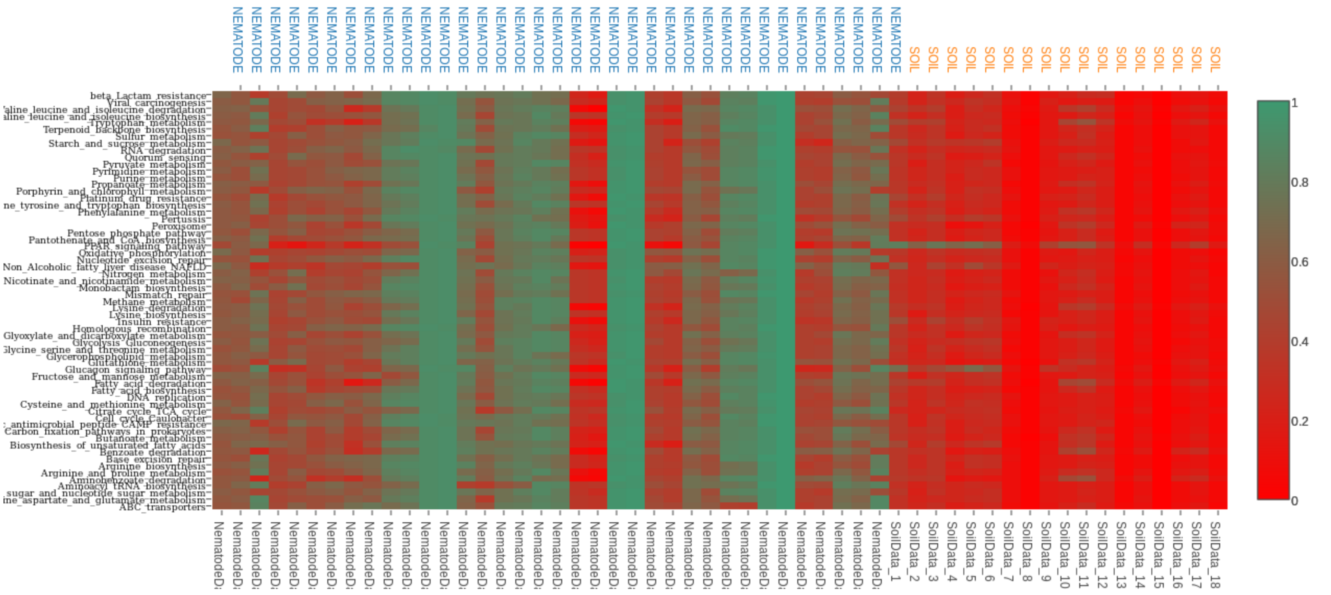

Figure SS9: Heatmap representing the (rank normalized) abundance profile of core functions inferred for Nematode and Soil metagenomic environments.

D. Differentiating Functions

Wilcoxon rank sum based differentiating function analysis was used by iVikodak for comparing the two metagenomic environments of Nematode and Soil. Following (Figure SS10) represents the cladogram of top differentiating functions.

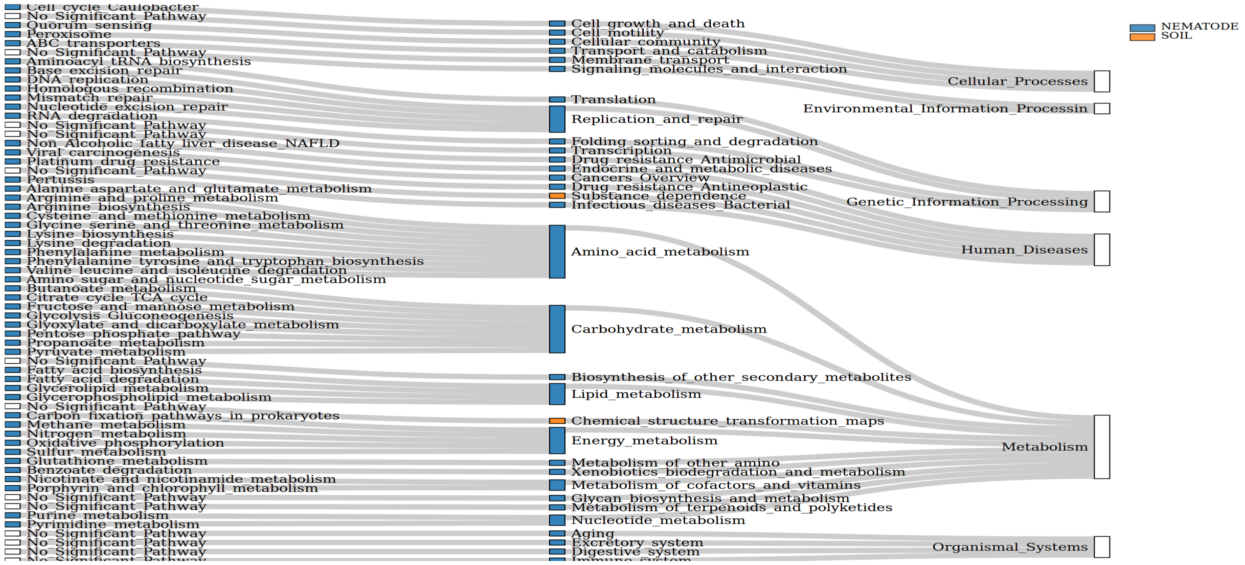

Figure SS10: Cladogram representing the differentiating functions between Nematode and Soil environments. Color of the node represents the environment in which the given function is significantly more abundant than the other.

E. Function driven networks

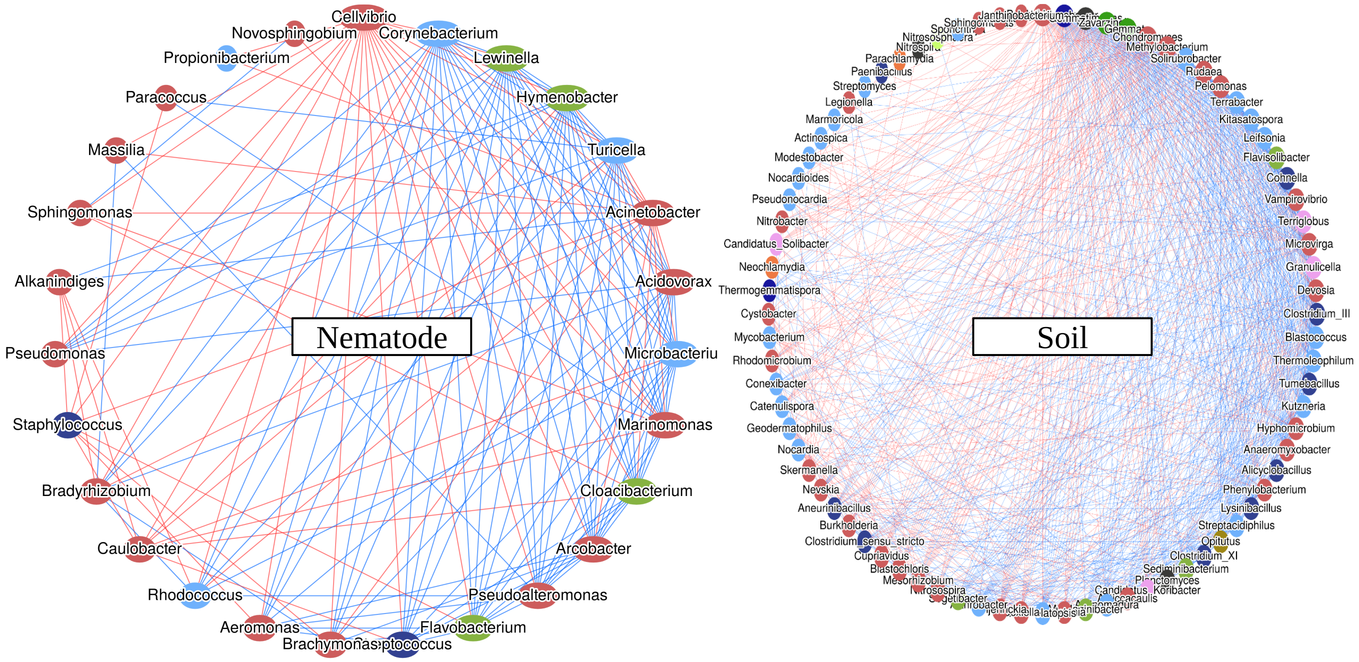

Figure SS11: *Function driven microbial interaction networks for Nematode and Soil environments. Color of the nodes represent their phylum affiliations. Networks are displayed in degree sorted circular layout.*
