# Supplementary material for: iVikodak—A Platform and Standard Workflow for Inferring, Analyzing, Comparing, and Visualizing the Functional Potential of Microbial Communities
Source: Front Microbiol. 2019 Jan 14;9:3336. doi: 10.3389/fmicb.2018.03336 (PMC6339920; doi:10.3389/fmicb.2018.03336)
Supplement: Supplementary file 4 [file Data_Sheet_4.pdf]

Supplementary file 4: Schematic representation of input and metadata formats

Taxa	S1	S2	S3	S4	S5	S6	S7	S8	S9	S10	S11	S12
Taxon-1	12	54	8	2	0	1	0	2	89	96	78	77
Taxon-2	0	0	6	3	4	5	5	3	12	32	23	31
Taxon-3	23	29	19	45	12	10	8	12	20	12	17	32
. .												
Taxon-n	1	0	3	1	101	189	90	78	88	56	62	49

Samples	Metadata-1	Metadata-2	Metadata-3		Metadata-n
S1	DDH	A20-30	Geo-1		Cat-1
S2	DDH	A20-30	Geo-1		Cat-1
S3	DDH	A20-30	Geo-1		Cat-1
S4	DDH	A20-30	Geo-1		Cat-1
S5	HHH	A30-40	Geo-2		Cat-2
S6	HHH	A30-40	Geo-2	.....	Cat-2
S7	HHH	A30-40	Geo-2		Cat-2
S8	HHH	A30-40	Geo-2		Cat-2
S9	AAA	Agt60	Geo-3		Cat-3
S10	AAA	Agt60	Geo-3		Cat-3
S11	AAA	Agt60	Geo-3		Cat-3
S12	AAA	Agt60	Geo-3		Cat-3

RDP ✔ GREEN GENES ✔ SILVA ✔
