# Supplementary material for: iVikodak—A Platform and Standard Workflow for Inferring, Analyzing, Comparing, and Visualizing the Functional Potential of Microbial Communities
Source: Front Microbiol. 2019 Jan 14;9:3336. doi: 10.3389/fmicb.2018.03336 (PMC6339920; doi:10.3389/fmicb.2018.03336)
Supplement: Supplementary file 5 [file Data_Sheet_5.pdf]

Supplementary File 5

A guide to choice of iVikodak's Modules

(Including usage guidelines/ SOP)

Objectives

1. Infer which pathways (and/ or pathway classes) are present (and in what proportion) in one or more samples from an environment of interest? → **Follow steps a, b and c given in SOP1**

2. Identify and visualize (inferred) functions that are highest in terms of abundance i.e. Top Functions (Box Plot) → **Follow steps given in SOP1**

3. Identify (inferred) "core" functions that are not only consistently present in samples constituting the up-loaded datasets, but also have a minimum abundance in the analyzed samples. (Visualize in form of Heat Map comprising of abundance of core functions in all uploaded samples) → **Follow steps given in SOP1**

4. Generate and visualize a network which depicts taxa correlated in terms of their contribution to the repertoire of functions in a specific environment? → **Follow steps given in SOP1**

5. Visualize a PCoA ordination plot in which samples are clustered based on the abundance pattern of inferred functions. Additionally, visualize (in form of a bar plot) the number/ proportion of samples in each of the clusters. → **Follow steps given in SOP1**

6. Overlay metadata corresponding to each of the samples in the visualizations generated in 2-6 above? → **Follow steps given in SOP1**

7. Identify and visualize functions whose abundance shows a statistically significant difference between the compared environments? → **Follow steps given in SOP1**

8. Perform statistical comparisons (Wilcoxon and (or) Kruskal-Wallis tests on a pair of or multiple environments) using the inferred function profiles (generated at various PEC thresholds using Global Mapper module). → **Follow steps given in SOP2**

9. Generate and visualize a heatmap of functions that are observed to be differentiating (at all PEC thresholds) between a pair of environments or multiple environments → **Follow steps given in SOP2**

10. Generate and visualize a Cladogram (visualized using Sankey plot), highlighting the hierarchical view of differentiating functions (at all levels) → **Follow steps given in SOP2**

11. Generate and visualize a Contributors Profile i.e. a filterable, sortable and exportable tabulated view of microbes that contribute towards the differentiating functions, along with their median contributions towards the functions in each individual environment and more. → **Follow steps given in SOP2**

12. Probe a specific function of interest. This function could be any function of interest. It could be a "Core Function" as identified using iVikodak's Global Mapper module. It could also be a function whose abundance pattern is identified by iVikodak's ISFA module as significantly different between two or more environments. → **Follow steps given in SOP3**

SOP 1

a. Navigate to iVikodak's "Global Mapper" Module

b. Upload "Taxonomic Abundance Table" i.e. your input file and "Metadata File", select appropriate parameters and 'Submit' job

c. For query 1, Download all results (in tab delimited format) from the Dashboard --> 'Downloads' tab --> Results.zip. The TSV files corresponding to various visualization in the Dashboard can be accessed using the following steps.

1. Extract contents of RESULTS.zip (downloaded from the Dashboards)
2. Access the directory named JobID.Results
3. Navigate to subfolder RESULTS → Analytics
4. The respective TSVs are available in folders named after the functionalities.

d. For queries 2-7, all results are provided as visualizations in a dashboard layout that enables interaction, layout edits, and download of publication quality images.

SOP 2

a. Navigate to iVikodak's "ISFA" Module

b. Upload a tab-separated file containing multivariate function abundance profile/ data obtained from Global Mapper; or a Functional.Profiles.at.PECs.zip obtained from Global Mapper. The former format is applicable for Rapid mode of operation while the later is applicable for Batch mode.

c. Upload corresponding "Metadata File", select appropriate parameters and 'Submit' job

d. Outputs are displayed in form of a Dashboard displaying results in form of -

1. PEC Profile: i.e. differentiating functions at various p-values viewed at various PEC thresholds in form of a heat-map (both at Level 2 and 3)

2. Cladogram of Differentiating functions at various p-values

3. Contributors to Differentiating Functions

SOP 3

a. Navigate to iVikodak's "Local Mapper" Module

b. Upload "Taxonomic Abundance Table" i.e. your input file and "Metadata File", select appropriate parameters and 'Submit' job

c. Outputs are displayed in form of a Dashboard displaying results in form of -

1. A heat map depicting Enzyme abundance profile (i.e.

2. A dendrobar depicting taxa contributing to that Function (The taxa are shown in context of their phylogenetic lineage)

3. KEGG data MAPS (3D Map file and a Color Map file) that can be downloaded and visualized by uploading to KEGG server mentioning the corresponding MapID

4. Corresponding textual files (tables) are also generated that can be downloaded from Dashboard --> 'Downloads' tab --> Results.zip. The TSV files corresponding to various visualization in the Dashboard can be accessed using the following steps.

1. Extract contents of RESULTS.zip (downloaded from the Dashboards)
2. Access the directory named JobID.Results
3. Navigate to subfolder RESULTS → Analytics
4. The respective TSVs are available in folders named after the functionalities.
