# Supplementary material for: iVikodak—A Platform and Standard Workflow for Inferring, Analyzing, Comparing, and Visualizing the Functional Potential of Microbial Communities
Source: Front Microbiol. 2019 Jan 14;9:3336. doi: 10.3389/fmicb.2018.03336 (PMC6339920; doi:10.3389/fmicb.2018.03336)
Supplement: Supplementary file 6 [file Data_Sheet_6.pdf]

Supplementary file 6: Multivariate Data analysis methods in iVikodak

Description of data analysis methods pertaining to inferred function data for deciphering Core Functions, Top functions, Differentiating functions and Function driven correlation networks are provided in this file

---

Index	
**Section**	**Access button**
**Core Functions**	Click to go
**Top Functions**	Click to go
**Differentiating Functions**	Click to go
**Function driven networks**	Click here
Note: There is a button on Top/ Bottom of each page to bring the reader back to this index.	

Core Functions

Core functions refer to those set of functions which appear consistently in most samples of a given metagenomic environment (e.g samples pertaining to Healthy individuals) at a minimum threshold of abundance.

In the context of iVikodak, Core Functions are computed using a bootstrapped approach as described below:

a. A random set of samples, equal to 75% of the total population size, is picked from the total population of samples pertaining to a given class of metagenomic environment.

b. Median abundance of all the inferred functions for the given environment is computed using the random set.

c. A minimum prevalence of 0.2 x Highest median abundance is set for core assignment.

d. For each function in the given randomly picked environment, assessment is performed for maximum prevalence proportion. Maximum prevalence proportion refers to the percentage of samples (in the random set) in which the given function had abundance greater than minimum prevalence.

e. All those functions are tagged as 'Tentative-Core' for the random set, which pass the prevalence proportion of 75%.

f. This process (between step a – e) is repeated 100 times and a boostrap score is assigned to all the Tentative Cores. Bootstrap score refers to the number of iterations in which a function appeared as

Tentative Core.

g. Those functions which have a minimum bootstrap score of 95% are tagged as Core Set of Functions for the given environment.

Visualization of the core functions is accomplished using a heatmap, which is generated using the rank normalized abundance profile of the union of core functions for all the classes of samples.

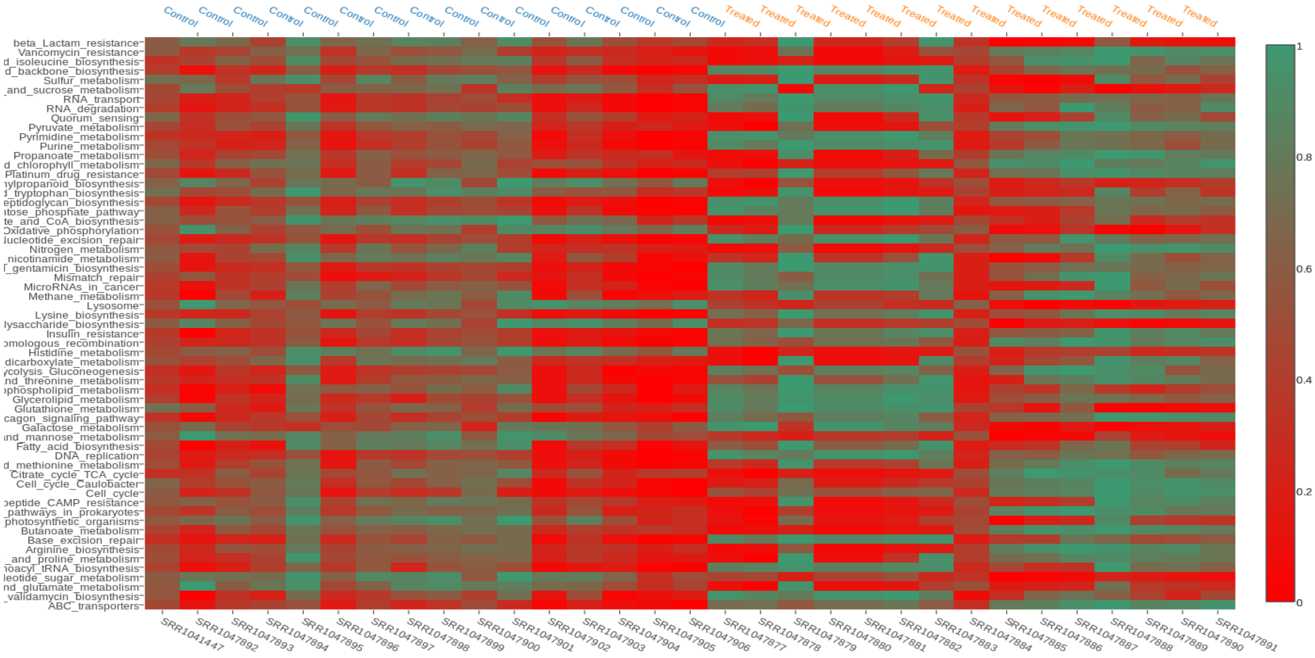

Figure SP1: Example of Core Function Visualization

Top Functions

Top Functions are computed using the median abundances of each inferred function in the environment. For the purpose of clarity, only Top 5 functions are selected from each class of samples in a population (e.g Top functions for Healthy class or Control class of samples).

Computation of Top Function set is done for Level 3 functions (most specific functional level, e.g Pyruvate metabolism) as well as for Level 2 functions (broader level in functional hierarchy, e.g Carbohydrate Metabolism).

Box plots are computed using the union set of functions deciphered for all classes of samples in the submitted taxonomic data.

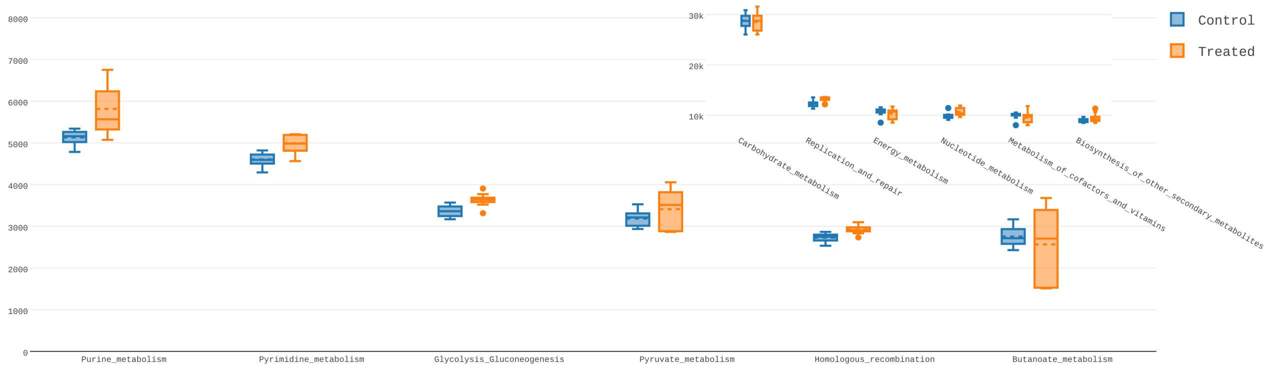

Figure SP2: Example of Top Functions Visualization (Main plot represents Level 3 functions, while inset represents Level 2 functions). Individual graphs for each level are also available in the dashboards

Differentiating Functions

Differentiating functions are those which can potentially act as signatures of distinction between multiple classes of metagenomic datasets (as supplied in the meta-data).

Differentiating features analysis is performed using classical tests:

1. Kruskal walis test (for multiple groups)

2. Wilcoxon rank sum test (for pair-wise differentiation)

As performed for the Top Functions, differentiating feature analysis is performed for both Level 3 and Level 2 functions are the hierarchy of differentiating functions is thereafter mapped to the Sankey based cladogram in the dashboard.

Uncorrected and BH corrected p-values are reported in the downloadable results, while the visualization module uses only BH corrected p-values.

As described in the main manuscript, Global Mapper module can infer functions using Pathway Exclusion Cut-off (PEC) thresholds. The ISFA module of iVikodak employs the functional inference at various PEC thresholds to arrive at differentiating functions of high-confidence. The Batch module of ISFA performs the statistical tests for the functional abundance matrices of all PEC values and then reports the PEC profile for each differentiating function. PEC profile is generated using binary information of 0/1 for each function, where 0 refers to absence of signature trait for a function at a given PEC value, while 1 refers to the presence of signature trait. A function which has signature trait at all or most PEC values is a potentially most significant differentiator.

Visualization of the PEC profile is accomplished using a heatmap, where green cells indicate presence of signature trait, while red cells indicate absence of signature trait.

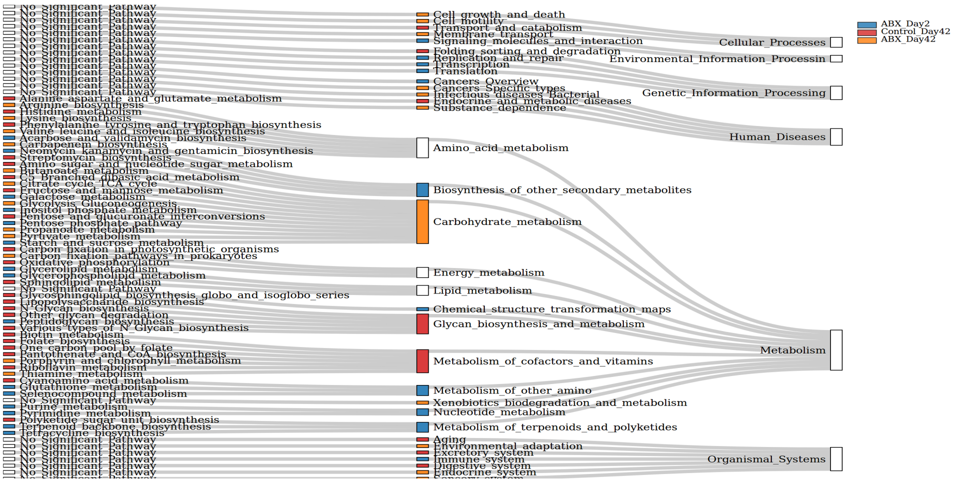

Figure SP3: Example of Cladogram view for differentiating functions. Color of nodes represents the class in which the differentiating function is most abundant

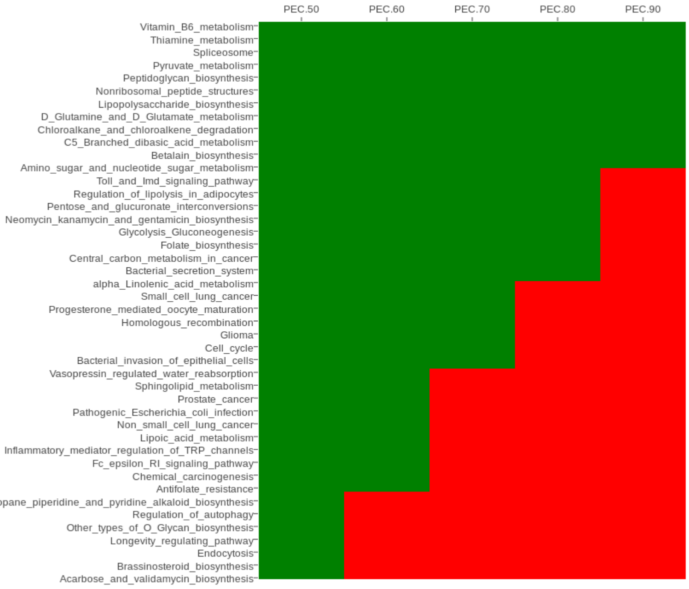

Figure SP4: Example of PEC profile for differentiating functions

Function Driven Correlation Networks

Vikodak introduced a novel concept of inferring inter-microbial interaction patterns using the correlations between their functional potentials (*Nagpal et al.*, 2016). iVikodak has realized that concept through automatic computation of correlations between contributing bacteria in a given environment. These networks are termed as 'Co-contribution' networks, because unlike co-occurrence networks derived from the abundance profiles of microbes in an environment, co-contribution networks, as detailed in Vikodak (*Nagpal et al.*, 2016), are derived from functional contribution profiles of resident microbes of an environment towards various inferred functions. iVikodak generates contribution profiles at all PEC values as well, thereby enabling the end users to probe function driven microbial interaction patterns using algorithms of choice. Visualization of such networks is enabled using Cytoscape.js and through the use of AJAX and in-house javascripting, various kinds of layouts are enabled for the end users, for all levels of meta-data.

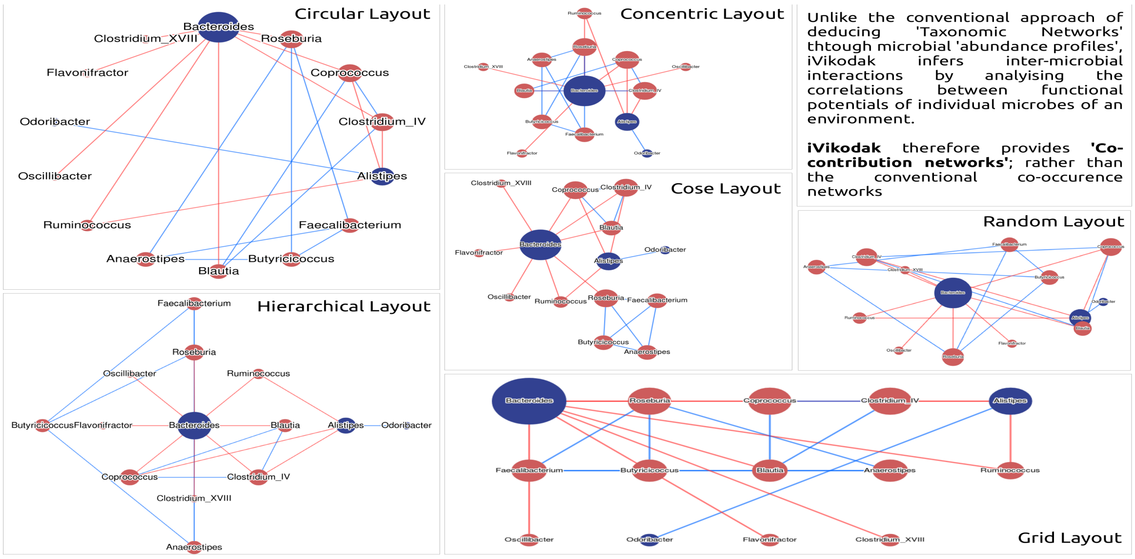

Figure SP5: Example of network visualizations possible in iVikodak
