# Supplementary material for: iVikodak—A Platform and Standard Workflow for Inferring, Analyzing, Comparing, and Visualizing the Functional Potential of Microbial Communities
Source: Front Microbiol. 2019 Jan 14;9:3336. doi: 10.3389/fmicb.2018.03336 (PMC6339920; doi:10.3389/fmicb.2018.03336)
Supplement: Supplementary file 7 [file Data_Sheet_7.docx]

**Supplementary file 7: Data availability reference**

Information regarding source of datasets used in this manuscript (for various case studies) has been provided in this file. Links to the abundance data matrices (and corresponding meta-data) for the said datasets have also been provided. The data matrices access forms a part of ReFDash database.

| **Case Study ID** | **Nature/ Site** | **Data Size** | **Reference**  **(Original Source)** | **Data matrix access link at ReFDash** |
| --- | --- | --- | --- | --- |
| 50a7bef1a5 | Oral  Cavity  (Human) | 91 | Griffen et al., 2012 | Abundance Data: [Access link](https://web.rniapps.net/iVikodak/Results/RESULTS/50a7bef1a5/50a7bef1a5.Results/Dashboard/Periodontitis.input.txt)  Meta-data: [Access link](https://web.rniapps.net/iVikodak/Results/RESULTS/50a7bef1a5/50a7bef1a5.Results/Dashboard/Periodontitis.MetaData.txt) |
| 998f4e89e5 |  |  |  |  |
| d819c619f7 |  |  |  |  |
| 6c32ef5cda |  |  |  |  |
| 6c32ef5cda | Vagina  (Human) | 394 | Romero et al., 2014 | Abundance Data: [Access link](https://web.rniapps.net/iVikodak/Results/RESULTS/6c32ef5cda/6c32ef5cda.Results/Dashboard/Human.txt)  Meta-data: [Access link](https://web.rniapps.net/iVikodak/Results/RESULTS/6c32ef5cda/6c32ef5cda.Results/Dashboard/MetaData_Body_sites.txt) |
| 6c32ef5cda | Skin  (Human) | 236 | Alekseyenko et al., 2013 |  |
| 6c32ef5cda | Gut  (Human) | 306 | Consortium THMP, 2012 |  |
| 6c32ef5cda | Gut  (Human) | 283 | Xiao et al., 2014  Kato et al., 2014 |  |
| 6c32ef5cda | Oral  Cavity  (Human) | 18 | Botero et al., 2014 |  |
| 6c32ef5cda | Oral  Cavity  (Human) | 55 | Cui et al., 2012 |  |
| 5cb3a79c2a | Soil | 18 | DDBJ ID: ERA411828 | Abundance Data: [Access link](https://web.rniapps.net/iVikodak/Results/RESULTS/5cb3a79c2a/5cb3a79c2a.Results/Dashboard/Nematode_Soil_abundance.txt)  Meta-data: [Access link](https://web.rniapps.net/iVikodak/Results/RESULTS/5cb3a79c2a/5cb3a79c2a.Results/Dashboard/Nematode_Soil_Metadata.txt) |
| 5cb3a79c2a | *Litoditis*  *marina* | 36 | DDBJ ID: SRP064694 |  |
